# Calcium Signals Driven by Single Channel Noise

**DOI:** 10.1371/journal.pcbi.1000870

**Published:** 2010-08-05

**Authors:** Alexander Skupin, Helmut Kettenmann, Martin Falcke

**Affiliations:** 1 Max-Planck-Institute of Molecular Plant Physiology, Potsdam, Germany; 2 Molecular Neuroscience, Max-Delbrück Centre for Molecular Medicine, Berlin, Germany; 3 Mathematical Cell Physiology, Max-Delbrück Centre for Molecular Medicine, Berlin, Germany; ETH Zurich, Switzerland

## Abstract

Usually, the occurrence of random cell behavior is appointed to small copy numbers of molecules involved in the stochastic process. Recently, we demonstrated for a variety of cell types that intracellular Ca^2+^ oscillations are sequences of random spikes despite the involvement of many molecules in spike generation. This randomness arises from the stochastic state transitions of individual Ca^2+^ release channels and does not average out due to the existence of steep concentration gradients. The system is hierarchical due to the structural levels channel - channel cluster - cell and a corresponding strength of coupling. Concentration gradients introduce microdomains which couple channels of a cluster strongly. But they couple clusters only weakly; too weak to establish deterministic behavior on cell level. Here, we present a multi-scale modelling concept for stochastic hierarchical systems. It simulates active molecules individually as Markov chains and their coupling by deterministic diffusion. Thus, we are able to follow the consequences of random single molecule state changes up to the signal on cell level. To demonstrate the potential of the method, we simulate a variety of experiments. Comparisons of simulated and experimental data of spontaneous oscillations in astrocytes emphasize the role of spatial concentration gradients in Ca^2+^ signalling. Analysis of extensive simulations indicates that frequency encoding described by the relation between average and standard deviation of interspike intervals is surprisingly robust. This robustness is a property of the random spiking mechanism and not a result of control.

## Author Summary

The number of proteins organizing cellular processes is huge. The challenge for systems biology is to connect the properties of all these proteins to cellular behavior. Do individual state changes of molecules matter for cell behavior despite these large numbers? Recently, we have experimentally shown for four cell types that intracellular Ca^2+^ signalling is driven by single channel dynamics. Molecular fluctuations are used constructively for a stochastic spike generation mechanism. The hierarchical structure of Ca^2+^ signalling prevents averaging of fluctuations and, consequently, the sequence of global spikes still reflects this molecular noise. Here we present a stochastic 3-D multiscale modelling tool living up to these findings by following the consequences of individual channel state changes up to cell level. We simulate the variety of cell responses in different experiments. The stochastic spike generation mechanism is surprisingly robust, providing new insights into the relation of function and robustness. The modelling concept can be applied to a large class of reaction-diffusion processes including other pathways like cAMP.

## Introduction

Cellular behavior is the dynamics emerging out of molecular properties and molecular interactions. Hence, cells are indispensably subject to intrinsic noise due to the randomness of diffusion and molecule state transitions in gene expression [1], [2], signaling pathways and control mechanisms. It drives noise induced cell differentiation [3], cell-to-cell variability of cloned cells [4] or second messenger dynamics [5]. While noise in gene expression can be attributed to small molecule numbers, we consider here noise in signalling pathways which occurs even in systems with large molecule numbers.

Molecular interactions create nonlinear feedback like substrate depletion and allosteric regulation in enzyme kinetics or mutual activation of ion channels in membrane potential dynamics. They also couple active molecules inside cells spatially by diffusion of product and substrate or electric currents. If this coupling is strong enough, cells respond spatially homogeneous. Otherwise, we observe dynamic spatial structures formed by concentrations of molecules in specific states. These structures are often called microdomains [6]–[9].

The existence of these dynamic structures determines in some systems whether the cell obeys deterministic or stochastic mechanisms. The dynamic compartmentalization of the cell by concentration gradients may prevent the establishment of deterministic dynamics by the law of large numbers even if the total number of molecules in the cell would suggest it otherwise. Microdomains are too small to behave deterministically. Not even the whole ensemble of microdomains will behave deterministically, if they are only weakly coupled or if there are only a few of them. Consequently, noise is not averaged out on cell level.

To determine whether we deal with a deterministic or stochastic system is important since these regimes may exhibit very different dependencies of behavior on system parameters [10]. For instance, repetitive spiking in intracellular 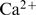 signalling would be restricted to parameter values providing oscillatory dynamics with a deterministic mechanism [11], [12]. It may occur with a stochastic system also for parameters which would lead to bistable or excitable dynamics in the deterministic limit, i.e. for larger or different parameter ranges [13]. In the non-oscillatory parameter ranges, the mechanism creating almost regular spike sequences can be coherence resonance [14]–[16] rather than the existence of a limit cycle in phase space of the local dynamics. Noisy systems with gradients usually show also a dependency of system characteristics on parameters of spatial coupling which spatially homogeneous systems do not exhibit. An example is the dependency of the spiking frequency on diffusion properties (see below and [5]).

In summary, the interaction between noise and gradients determines parameter dependencies and mechanisms. Recent experimental and theoretical studies on intracellular 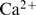 dynamics taught us that cells may indeed work in this regime and may exhibit repetitive spiking with non-oscillatory local dynamics. Functionally relevant gradients are also observed with intracellular cAMP [8], [17]–[19], pH [20] and in phosphorylation/dephosphorylation dynamics [21], [22] suggesting that the lessons learned from 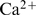 dynamics may also apply to other systems.

One of these lessons is that the randomness of single molecule state changes is carried up from the molecular level to cell level [23], [24]. Cellular 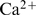 concentration spikes form random sequences of interspike intervals (ISIs) and that randomness arises from the randomness of single molecule state transitions [5], [25]. Consequently, the fluctuations of cellular signals contain information on single molecule behavior. It is a task for modelling now to establish the relation between these fluctuations and single molecule properties to decode this information.

Systems exhibiting the interaction between noise and gradients require modelling tools which can deal efficiently with the large concentration gradients and with the time scale range from molecular transitions to cell behavior. Here, we present such a modelling concept with the example of intracellular 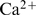 dynamics. It simulates all active molecules as stochastic Markov chains with all the individual state transitions and describes diffusion and some bulk reactions deterministically. Active molecules are those carrying the crucial feedbacks and nonlinearities. That allows for linearization of passive bulk reactions and the application of a multi-component Green's function to solve the partial differential equations in the cell analytically. We combine Green's functions with a local quasi-static approximation for the fast concentration changes and diffusion processes at the location of active molecules. That is possible due to the short diffusion time on the molecular length scale of a few nanometers. Since we use Green's functions for the long range concentration profiles we can restrict the calculation of concentration values to the location of active molecules. That renders this method extremely efficient even in 3 spatial dimensions.

We will apply this concept to intracellular 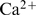 dynamics and compare simulated time dependent concentrations with single cell time series obtained from cultured astrocytes all measured under the same condition without any stimulation. 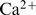 is a ubiquitous second messenger in eukaryotic cells that transmits a variety of extracellular signals to intracellular targets. 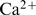 controls fertilization, cell differentiation, gene expression, learning and memory [26]. It triggers secretion in glands, muscle contractions in the heart and transmits apoptosis signals [27], [28].

A main mechanism to increase the cytosolic 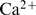 concentration is release from intracellular stores, especially from the sarcoplasmic reticulum by ryanodine receptor channels (RyRs) or the endoplasmic reticulum (ER) by inositol 1,4,5-trisphosphate receptor channels (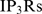). These channels open in a 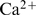 dependent fashion - a self amplifying effect known as 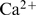 induced 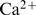 release (CICR) [27], [29]. If a single channel opens, 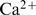 is released into the cytosol, diffuses to adjacent channels and increases their open probability. Thus release may spread into the entire cell leading to a global cytosolic 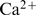 concentration spike.

**Figure 1:**
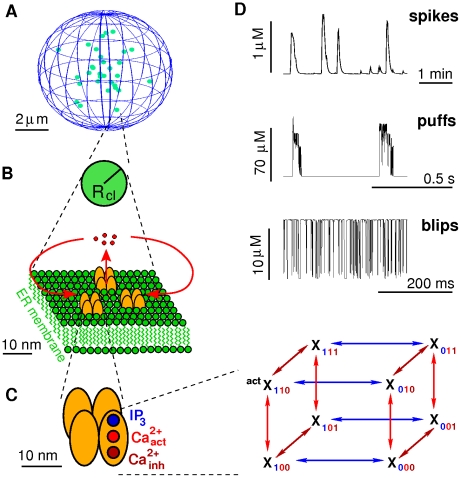
IP_3_R properties and clustering generate a hierarchical system. **A**: 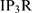 form channel clusters (green dots) that are randomly scattered across the membrane of the ER and separated by 1 to 7 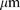 in the cell. **B**: Compared with inter-cluster distances, channels (orange) within a cluster are tightly packed in the ER membrane and are strongly coupled by 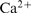 (red). Channels within a cluster are lumped into one source term (green sphere) with radius 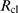, which depends on the number of open channels (see text). **C**: Single 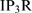 consist of four subunits the dynamics of which is described by the DeYoung-Keizer model. The 8 subunit states form a cube and subunit state transitions correspond to the edges. **D**: The 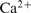 dependent activation and inhibition of 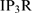 are key elements of 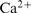 induced 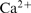 release. Combined with the spatial clustering, the resulting hierarchical structure transforms fast fluctuating single channel dynamics (blips) first into locally amplified cluster signals (puffs) and then into cellular release spikes. (Local concentrations are determined 10 nm apart from the release site.)


The inositol 1,4,5-trisphosphate (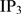) pathway initiates 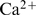 release from the ER in many cell types (including astrocytes [30]), since binding of 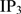 to the 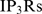 primes them for activation by 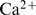 (Figure 1 in Text S1). The spatial arrangement of 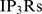 in channel clusters leads to a hierarchical system with the structural levels channel, channel cluster and cluster array, which is the cell level. 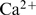 pumps and buffers generate large gradients close to open channel clusters. Thus, channels within a cluster are strongly coupled and the coupling between clusters is only weak - the geometrical hierarchy entails a hierarchy of coupling strengths.

Stochastic binding of 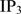 and 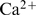 to the binding sites of 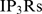 leads to random opening of a single channel in a cluster [31], [32]. This causes other channels of the same cluster to open also leading to a puff. An individual cluster is stochastic due to the small number of 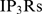 per cluster [33]–[35]. The opening of a single cluster can only be detected by adjacent clusters due to the strong 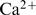 gradients [23], [24], [27], [36], [37]. Since they are again only a few, it remains random whether they are opened by the initial puff. If a supercritical number of puffs arises, release spreads into the whole cell causing a global spike. Thus, due to the hierarchy of coupling strength, randomness is carried up from the channel level to the cell level.

In order to model the hierarchical system, we have to consider the stochastic behavior of individual 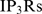 and the spatial heterogeneity of cells induced by 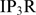 clustering. That leads to a reaction diffusion system (RDS) with local stochastic source terms. For sufficient fast simulations, we decompose the system into local stochastic dynamics comprising channel state transitions and fast local concentration changes and a deterministic global dynamics for which we derive an analytical solution in form of a three component Green's function (Text S1). The solution is driven by stochastic channel behavior described by a hybrid deterministic-stochastic algorithm. We apply the model to a variety of experiments to demonstrate its potential.

## Results

### Multi-scale modelling exploiting the hierarchical organization of Ca^2+^ signals

Our modelling concept simulates active molecules individually by Markov chains, the concentration dynamics in the range of the molecule locally quasi-statically and the diffusional long range coupling by Green's functions. Simulations are orders of magnitude faster than numerical schemes based on spatial grids. Their efficiency derives from the methods which we apply. The use of hybrid deterministic-stochastic algorithms for the Markov chains allows for time steps much larger than traditional Gillespie algorithms. In between stochastic molecule state transitions, we integrate the concentration dynamics. The local quasi-static approximation reduces clusters to spatial 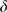-function sources which turns integrals into sums. It also substantially reduces the number of modes to be used in the Green's function. And finally Green's function enables us to restrict the calculation of concentration values to the locations of active molecules.

### Channel and cluster level


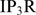
 dynamics and spatial channel clustering lead to the hierarchical system depicted in Figure 1. 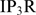 channels are tetrameres [38]. A single channel opens and closes in dependence on binding and dissociation of 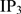 and 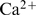 to the binding sites of its subunits (see below). An open channel conducts a 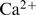 current from the ER into the cytosol which is due to the huge concentration difference of up to 4 orders of magnitude across the ER membrane.

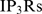
 form clusters on the membrane of the ER consisting of 1 to 10 channels [33], [35]. They physically interact within a cluster and are consequently separated by a few nanometers only [35]. The 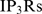 in a cluster are strongly coupled by the large local 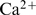 concentration close to open channels.

Typical inter-cluster distances found experimentally are in the range of 1–7 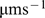 [39]. Figure 1A shows a representative example of cluster arrangement used in simulations. Due to cytosolic buffers and SERCAs, the local 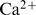 concentrations close to an open channel cluster exhibit large gradients such that coupling between clusters is weak compared to intra-cluster coupling. This leads to the hierarchical organization of 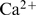 signals. Stochastic opening of a single channel (blip) is locally amplified by CICR leading to a puff (Figure 1B and D). The concentration gradients keep the probability for activation of adjacent clusters small and only a fraction of puffs activates several neighboring clusters. Once a supercritical number of open clusters is reached, more of them open forming a global signal. In that way, the triggering random opening of a single 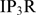 is carried up to the macroscopic scale. The mechanism transforms the fast noise of channel state changes on a millisecond time scale into fluctuations of interspike intervals of tens of seconds as shown in Figure 1D.

An early and widely used channel state model is the DeYoung-Keizer model [40], [41]. It assumes independent subunit dynamics and allocates three binding sites to each subunit as shown in Figure 1C. One site for 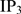 and one for 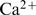 that cooperatively activate the subunit. Another binding site with lower affinity for 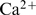 inhibits the subunit dominantly. These two different affinities lead to a biphasic dependence of the stationary open probability on the 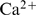 concentration (see Figure 1 in Text S1). Only the state 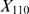 out of the 8 possible subunit states 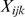 corresponds to an active subunit (Figure 1C), where the first index refers to the 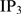 binding and is 1, if 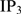 is bound and 0 otherwise. Analogously, the second and third index describe 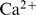 binding to the activating and inhibiting site, respectively. A channel opens, if at least 3 subunits are in the active state.

The 12 possible transitions between the 8 subunit states correspond to transitions in a state scheme forming a cube (Figure 1C). Some of the transition probabilities depend on the local 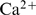 and 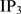 concentrations (Figure 1 in Text S1). In simulations, the transitions are realized by a hybrid deterministic-stochastic algorithm [42], which uses the local 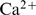 concentrations and the dissociation rates and binding rate constants given in Table 1 in Text S1.

Since 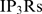 within one cluster are close to each other, a cluster can be approximated by one spatial 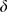-source for the purpose of simulating the cluster current in the long range cellular dynamics. The current depends on the number of open channels 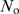, the time course of which comes out of the stochastic simulation of channel states. It is proportional to the concentration difference 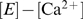 across the ER membrane at the location of the channel molecule. Hence, we actually need to solve the complete reaction-diffusion problem to determine it. But the concentration difference at the cluster is not well defined with a 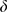-source term. Therefore, we calculate the cluster current using a spatially extended cluster with radius 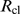 as described in detail in Ref. [43]. The solution of that problem converges within fractions of a millisecond to its stationary state in the range of the channel molecule [43]. That part of the solution is all we need to calculate the current of the 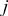
th cluster. Using the stationary concentration profiles we obtain:
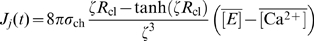
(1)with 
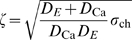
 where 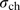 denotes the channel flux constant. 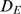 and 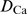 are the diffusion coefficients of 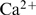 in the ER and the cytosol. The cluster radius 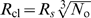 depends on the number of open channels 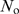 and the single channel radius 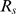. The advantage of the approximation is that it takes local ER depletion into account but only depends on the the spatially averaged concentrations 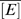 and 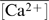, which form the boundary conditions for the local quasi-static approximation (see [43] for details). If channel distances within a cluster are of the order of magnitude of the diffusion length of free 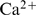, the internal cluster geometry becomes relevant. In that case, several 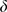-functions can be used for one cluster.

The approximation allows as well for determination of the local 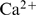 concentration at an open channel cluster resulting from its own current (1) as
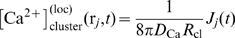
(2)the validity of which had been shown for the buffer concentrations used here [43]. Note that the total concentration at a cluster is the sum of the concentration (2) and the concentrations induced by currents of other open channel clusters. After closing, the 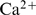 concentration is determined by the cellular concentration dynamics (see below) 10 nm apart from the release site.

### Cellular concentration dynamics

The modelling strategy for the cellular 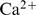 dynamics is based on the separation of two length scales. On the microscopic scale of channel clusters, we use a detailed and stochastic channel model to determine local 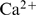 currents. On the macroscopic scale of the cell, we use a linearized spatial bi-domain model, and Green's function to integrate it. The microscopic scale determines the currents representing the 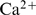 sources of the macroscopic scale. We implement ideas proposed in [43] and use the currents 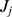 of Eq. (1) as the amplitudes of the spatial 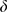-functions representing the cluster source terms in Eqs. (3). A similar approach was taken by Solovey *et al.* [44]. We circumvent the concentration divergence at 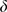-function sources by using Eq. (2) for the value of the local concentration at open clusters. Vice versa, the macroscopic scale affects the concentration values entering the transition rates of the microscopic state schemes.

The ER is a tubular network spreading throughout the cell [45]. Diffusion in such a geometry can be described by diffusion in unrestricted space with a decreased diffusion coefficient [46]. Subsequently, we can superimpose the ER and the cytosol leading to a bi-domain model. Due to the quasi-static approximation (Eq. 1), we do not need to determine the spatially resolved concentration in the ER. Lumenal and cytosolic domains are coupled by a homogeneous 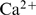 leak flux 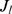 through the ER membrane, 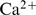 re-uptake 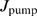 of the ER by SERCA pumps and by the stochastic channel currents 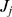. Within the cytosol we take free 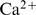, one mobile buffer 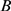 and one immobile buffer 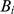 with the total concentrations 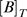 and 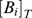 into account leading to the reaction diffusion equations
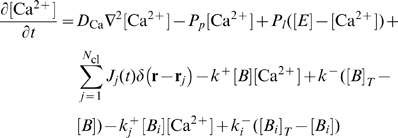
(3a)

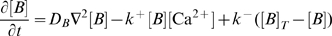
(3b)

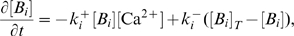
(3c)where we used buffer conservation and linear pump and leak fluxes with the flux constants 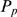 and 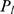. 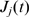 is the stochastic channel cluster current of the 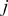
th cluster with strength defined by Equation (1).

**Figure 2:**
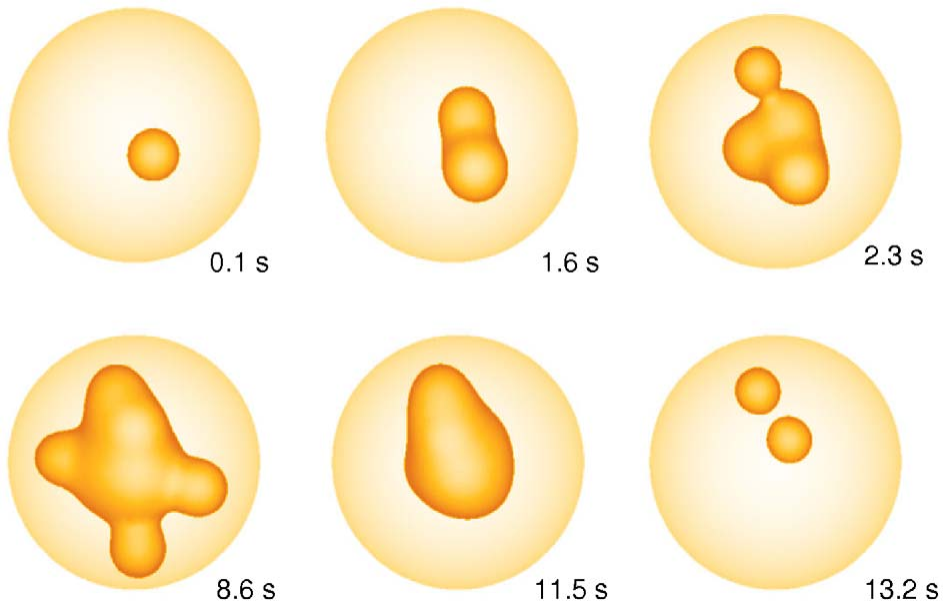
Spatially resolved Ca^2+^ dynamics. An initial puff induces 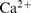 release of adjacent clusters by diffusion and 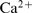 induced 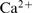 release leading to a global 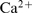 spike. The puff to spike transition is visualized by the iso-concentration surface of 2 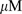 during a spike. Time is indicated on the panels (see Video S1).

Scaling concentrations, space and time with typical values reveals the number of independent parameters. It entails the definitions of Table 2. We linearize Eqs. (3), since we would like to use Green's function to solve them. Our parameter values are in the range of the applicability of the linearization to the buffer dynamics as described by Smith *et al.* [47] for the stationary profiles. We additionally have linearized the pump dynamics. The linearization does not exhibit saturation, which is relevant for calcium concentrations above 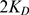, with 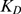 being the dissociation constant of the pump (Figure 2 in Text S1). These concentrations occur close to open clusters. In that area, the dynamics are dominated by the diffusion term and the channel term, which reduces the relative error due to the linearization of pump and buffer rates substantially. However, if precise knowledge of concentration values close to open channels or clusters is required, the complete non-linear reaction diffusion equations must be solved like e.g. in [42]. The scaled linear reaction diffusion system (Text S1) describes the spatially resolved concentration dynamics by:
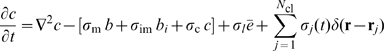
(4a)

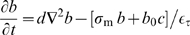
(4b)

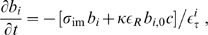
(4c)where the leak flux depends on the average lumenal concentration, only. All the reaction rate constants depend on the resting state concentration 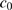, 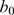 and 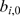 due to the linearization: 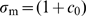, 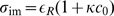 and 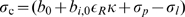. For simplicity we subsumed also 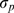 and 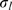 under 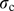.

**Figure 3:**
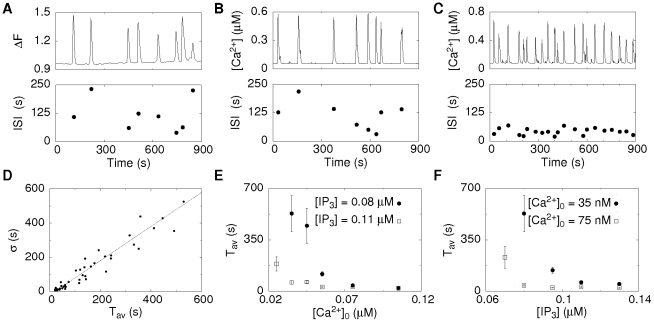
Stochasticity of Ca^2+^ oscillations. **A**: An experimental example of 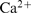 oscillations in an astrocyte. The varying ISIs demonstrate the stochasticity of spiking. **B,C**: Simulations of the cellular 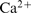 dynamics of a cell with 47 clusters each having a random number of channels between 4 and 16 for different 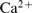 base level 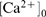 concentrations and the standard parameters given in Table 1. For a low 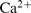 base level of 30 nM spiking is rather slow and irregular (B). For an increased 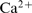 base level of 50 nM spiking becomes faster and more regular (C). **D**: The simulated 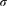
−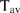 relation, where dots correspond to spike trains of single cells having different 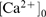 and 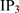 concentration (see Figure 5 in Text S1), is in accordance with the experimentally observed one [5] supporting the wave nucleation mechanism. **E,F**: The dependence of the average period 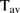 on the 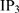 concentration and the 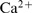 resting concentration obtained in simulations show that regular spiking is more likely if one concentration is high.

The cytosolic concentrations 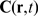 are determined by the 3-component Green's function with 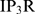 clusters localized at 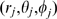 (see also Figure 3 in Text S1)
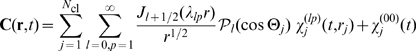
(5)with the Bessel function of the first kind 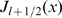 and the Legendre polynomial 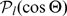, where 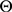 is the angle between the source location 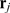 and the point 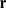 given by
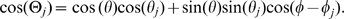
(6)


The 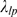 are determined by the boundary conditions at the plasma membrane (see Text S1).

The three-component response functions 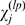 and 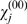 include the time integration over the source history, i.e. the time dependent channel flux strength 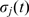, and take the buffer reactions as well as the coupling with the ER into account:
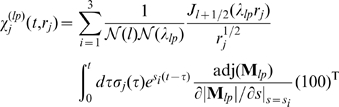
(7a)

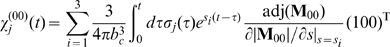
(7b)with the dimensionless cell radius 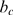 and the normalization factors 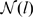 and 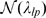 given in the Text S1. The coupling between the cytosol and the ER by 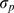 and 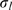 as well as the reaction rates of 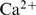 with the two buffers determine the time constants 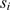 of the response functions (0), which are implicitly given by the roots of the determinant of the coupling matrix
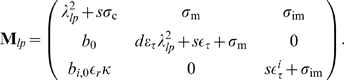
(8)


The method allows for spatially resolved concentration dynamics as shown in Figure 2 and in the Video S1 by an iso-concentration surface of 2 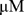. An initially opening cluster increases the open probability of adjacent 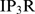 clusters and release is spreading through the cell until inhibition stops release.

For the global 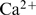 dynamics, the average concentrations are obtained by spatial integration of the analytical solution (9) as
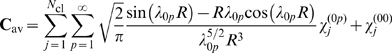
(9)where 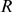 denotes the cell radius. The first component of 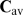 describes the cytosolic average concentration 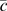. With this, the lumenal average 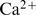 concentration 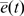 in dimensionless units is determined by
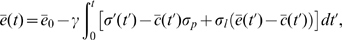
(10)which takes into account the leak, pump and channel fluxes, and 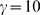 is the volume ratio 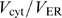 of the cytosol and the ER. 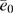 denotes the equilibrium average lumenal concentration at 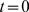. The difference between the average cytosolic and lumenal concentration 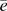
−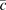 determines the cluster current according to Eq. (1) (see Text S1).

**Figure 4:**
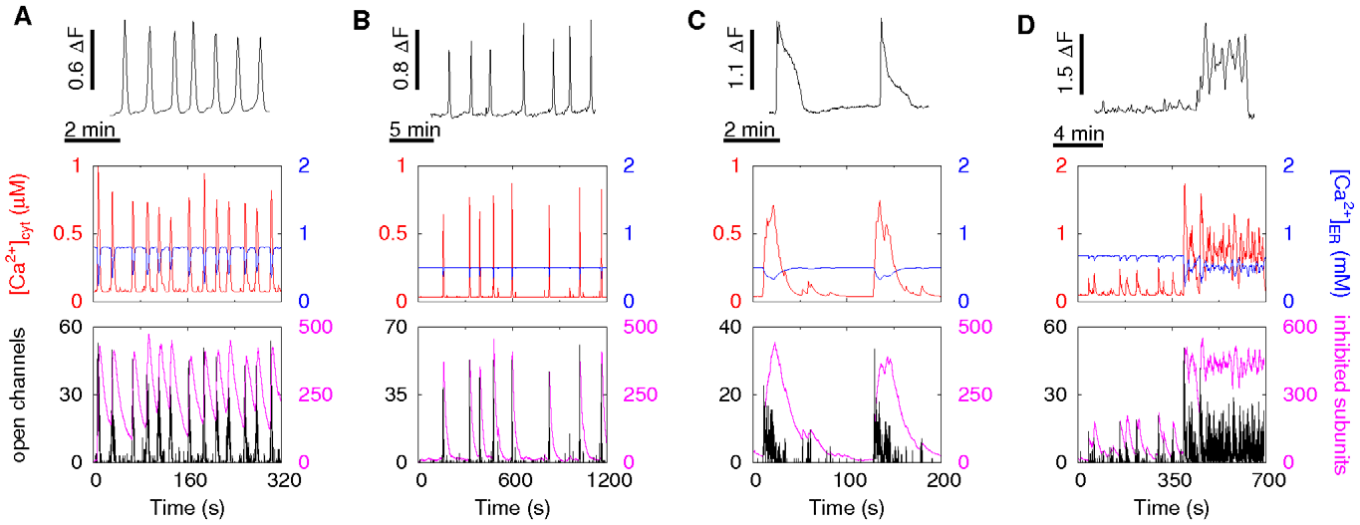
Spontaneous Ca^2+^ signals in individual astrocytes measured under identical conditions (upper row) and simulations of a cell with 32 clusters with different parameters (red line, middle row) exhibit good agreement in the cytosolic Ca^2+^ concentration. The parameter changes between the simulations account for the variability of the cells in the experiment. The lumenal concentration is shown in blue (middle row). The channel dynamics (lower row) is shown as the number of open channels (black) and inhibited subunits (magenta). **A**: Fast and regular spiking occurs by array enhanced coherence resonance where the simulated cell spikes as soon as enough channels are in the excitable state again. Spikes occur before the cell reaches its resting state as can be seen from the time course of the fraction of inhibited subunits. This is caused in simulations by a high 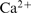 base level concentration 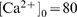 nM and a 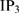 concentration of 0.12 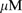. **B**: Spontaneous oscillations exhibit often a more irregular spiking. This is achieved in simulation for the same cellular setup as in A by a 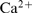 base level concentration of 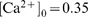 nM, which is lower than the standard value of 50 nM (Table 1). That decreases the probabilities for an initial event and spikes compared to panel A. The cell reaches the resting state before some of the spikes. **C**: A bursting like behavior is observed for decreased SERCA activity (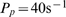) in simulations, since 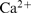 remains longer in the cytosol. **D**: For a even smaller SERCA activity of 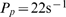, 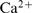 signals obtained in simulations exhibit plateau responses with superimposed oscillations which are also found in experiments. Simulation parameters are given in Table 1 if not stated here.

The two main approximations of our method are the local quasi-static approximation and the linearization of the passive bulk processes. These assumptions do not allow for a precise study of the intra-cluster concentration dynamics. That can be done with finite element methods like in ref. [42]. The structure of the Green's function solution enables an elegant parallel algorithm that we call the Green's cell. It is orders of magnitude faster than finite element methods and able to simulate long lasting whole cell dynamics in feasible computing time. In the Green's cell algorithm the actual concentration of each cluster is calculated with the Green's function and local quasi-static approximation in dependence on the source history of all clusters by a single process. The concentrations are sent to the master process which determines the corresponding state transition and reaction time by the hybrid algorithm and also calculates the average concentrations. The transition times are re-distributed to the cluster processes where they are used to update the concentrations. For further details see Figure 4 in Text S1.

### Stochasticity in measured and simulated Ca^2+^ signals

Our recent experimental investigation started from the assumption of a random spike generation by wave nucleation followed by a deterministic refractory time. This prediction yields in a linear dependence of the standard deviation on the average period which was also experimentally confirmed [5]. Previous studies report a possible feedback of 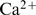 on PKC activity in glutamate stimulated rat astrocytes [48]–[50]. This may lead to a positive feedback on the 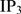 level by activation of PLC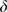. The measured relation between standard deviation and average of interspike intervals for spontaneous spiking has a slope equal to 1 [5], demonstrating that spike generation is poissonian and the spike generation probability is constant on the time scale of ISI. Clearly, there is no feedback on that time scale.

To show that the experimental findings are indeed consistent with our ideas of spike generation, we use our modelling tool to study how molecular noise of single channels can be translated into global signals and whether it is sufficient to cause the observed randomness of spike sequences. Figure 3A shows an example of single cell measurements, where the upper panel exhibits the fluorescent signal 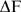 related to the cytosolic 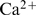 concentration and the lower panel the individual ISIs. It demonstrates the stochasticity of spiking, since variations in ISIs are in the range of their average. Simulations of a cell with 47 clusters each containing a random number of 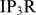 between 4 and 16 exhibit a behavior very similar to experiments showing that single channel noise can lead to time varying ISIs, since there are not any other sources of noise in the simulations (Figure 3B and C). The simulated 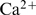 oscillations exhibit in accordance with experimental observations different flavors ranging from rare and irregular spiking to fast and more periodic spiking. The standard deviation 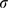 depends linearly on the average period 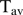 [5]. Recently we have shown that this linear dependence is not a self-evident relation [51]. In particular, it was found that self-sustained oscillatory systems exhibit a different relation than the one observed in 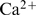 spiking experiments. The dependence of 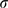 on 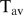 obtained here in simulations is shown in Figure 3D and exhibits a linear dependence with a slope of 1 which was found in experiments for spontaneous oscillations [5], [52]. The offset of the regression line on the 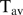 -axis of about 20 s is the deterministic recovery time.

### Dependence on IP_3_ and Ca^2+^ concentrations

**Figure 5:**
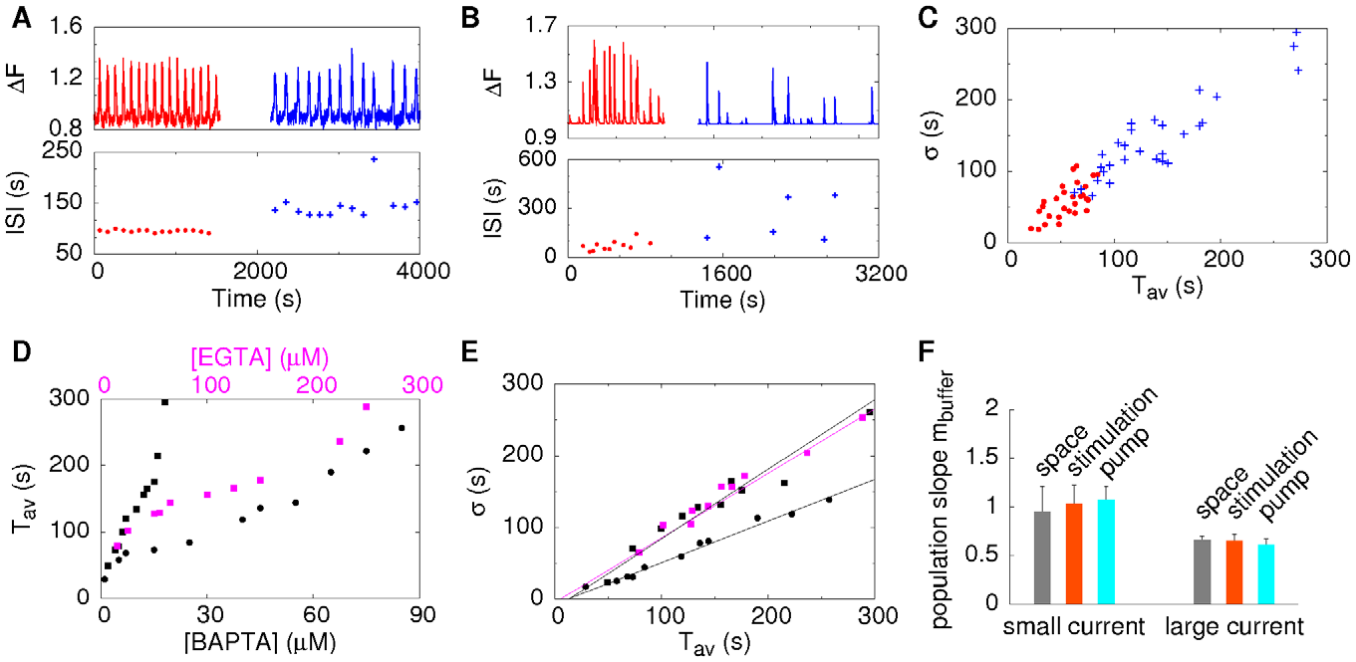
Buffers render spiking more irregular by decreasing spatial coupling. **A**: Astrocytes were measured several minutes for reference values (red) before loading with 20 nM BAPTA-AM during the break and restarting the measurement (blue). Fast and regular spiking is shifted to a slower and more irregular one. **B**: Simulation of a cell containing 32 clusters with two different EGTA concentrations shown in red and blue respectively exhibit an analogous behavior. **C**: An increase of 10 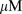 EGTA increases 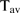 and 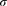 for a population of simulated cells with different cell properties, very similar to experimental observations. **D**: 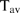 increases with increasing EGTA (magenta) and BAPTA (black) concentration for a given cell. The value of the increase depends on the single channel current. Squares correspond to 0.12 pA and dots to 1.2 pA. **E**: Corresponding 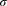
−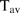 dependence of simulations in panel D. BAPTA and EGTA lead to a similar 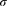
−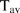 dependence for the smaller current (squares), whereas the increased current decreases the slope to 0.6. **F**: A single channel current of 0.12 pA leads to a population slope 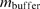 of 1 rather independent of spatial arrangement of clusters (gray), stimulation strength (light red) and pump strength (light blue) where the population slopes arise due to 10 different buffer concentrations (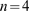 simulations for each condition). For the larger current of 1.2 pA the slope decreases to 0.6 and is again relatively independent of other physiologic parameters. This may explain the experimentally observed cell specific slopes [5]. Parameters used in simulations are given in Table 1 if not explicitly stated here.

The different 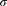
−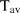 data points in Figure 3D result from different combinations of the 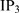 and 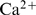 base level concentrations, which are both parameters in the model. *In vivo* the 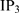 concentration is related to the stimulation level by activation of Phospholipase C and 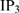 production. The 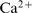 base level is determined by the leak and the pump flux through the ER membrane. In simulations, we adjust the leak flux according to 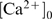 and the pump strength. If both concentrations are rather high in the range of 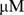 no spiking occurs since channels are activated as soon as they are in the excitable state (Figure 5 in Text S1). We observe fast and regular spiking (Figure 3C,E and F and Figure 5 in Text S1) for intermediate concentrations. The ISIs have a 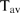 close to the deterministic refractory time, since a new spike is initiated as soon as the recovery time has elapsed. Regular spiking corresponds to cells with small 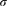 in Figure 3D. A further decrease in one of the concentrations increases 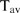 and 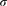, in a way depending on the other concentration (Figure 3B,E and F). If both concentrations are small, global spiking vanishes and the signal consists of uncorrelated blips.

### Different Ca^2+^ signals in dependence on physiologic parameters

In the previous analysis of the dependence of oscillations on the concentrations, we have already seen that the modelling tool can generate a large spectrum of 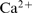 signals ranging from stochastic spiking to almost periodic oscillations. Here, we show that the model can produce all known 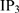 -induced forms of 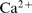 signals in dependence on physiologic parameters. Figure 4 exhibits different experimental signal forms and the corresponding simulation results for a cell with 32 clusters. The variety of signals is achieved by varying cell parameters leading to distinct cell responses as shown by the behavior of open channels (black) and number of inhibited subunits (magenta) as well as by the resulting average 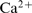 concentration in the cytosol (red) and in the ER (blue). Fast and rather regular oscillations occur by the interplay of activation and inhibition leading to array enhanced coherence resonance as was hypothesized before [5]. This can be seen in the behavior of the channel state dynamics. The number of inhibited subunits (magenta) increases dramatically during a spike and finally inhibition terminates it (Figure 4A). In the following the number of inhibited subunits relaxes slowly towards its resting level. Only very few channels open directly after a spike and these openings do not initiate a new spike, since the number of inhibited subunits is still to high (higher than approximately 220). That causes the deterministic time 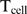 also observed experimentally [5], [52]. But a spike occurs very soon after the number of inhibited subunits has fallen below a critical range since the open probability is rather high with these parameter values. That keeps the stochastic part of the ISI small and spike sequences regular. Moreover, the amplitude of the spike of open channels seems to be smaller, if the spike is initiated at times where the number of inhibited subunits is still high.

We find longer and more irregular ISIs for decreased 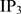 and 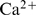 base level concentrations, since the probability of a channel opening is decreased. As a consequence, the cell relaxes to a resting state between spikes with only a few inhibited subunits (Figure 4B). The spike amplitudes of both the number of open channels and of the average 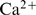 concentration are slightly increased compared to the regular spiking.

SERCA pumps also shape 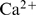 signals. Recent studies have shown that different phenotypes of cloned cells with regard to 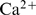 signalling occur due to small variations in SERCA expression levels and activity of RyR [4]. Here, we find that a decreased SERCA activity leads to a burst like behavior (Figure 4C), since 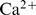 is removed slower from the cytosol and thus can activate channels which have recovered early from inhibition or channels which have not been activated before.

For even smaller SERCA activity, cells exhibit long lasting plateau 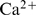 signals often with superimposed oscillations (Figure 4D). In these cases, released 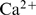 stays within the cytosol and reactivates 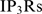 again and again. Cooperativeness induced by inhibition leads to superimposed oscillations on the high 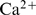 level. The panels of Fig. 4 provide also an idea of cell variability within one cell type and even within one experiment.

### Increased randomness by Ca^2+^ buffers

A direct consequence of the diffusion mediated signal mechanism is the dependence on the strength of spatial coupling by 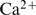 diffusion. That coupling strength can be modulated by exogenous 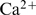 buffers, since they reduce the diffusion length of free 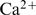. We took advantage of this property of buffers to demonstrate the spatial character of 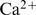 oscillations [5]. Note that we used concentrations of 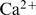 buffers much smaller than usually applied in order to suppress any kind of 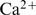 signal. We measured spiking for several minutes to obtain reference values for ISIs, loaded additional buffer and continued measuring (see Figure 5A). The individual ISIs (blue crosses) are increased and exhibit a larger variability after buffer loading.

To understand the experimental observation in more detail, we use simulations to analyze the response to additional buffer. Analogously to the experiment, we simulate a fixed cellular arrangement with different mobile buffer concentrations. Figure 5B shows a representative example, where the red and the blue parts correspond to 25 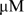 and 250 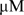 EGTA, respectively. Like in the experiment, larger buffer concentration leads to less and more irregular spiking. In the part with the higher buffer concentration, we observe isolated events which do not lead to global waves since coupling of clusters is too weak. These local events are rare in the reference measurements, since a triggering event initiates a global wave very likely.

From population simulations, where individual cells differ in their spatial arrangement of clusters, initial buffer and 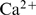 base level concentrations, we obtain the 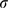
−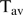 relation shown in Figure 5C, where cells are shifted by an increase of 10 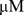 in the EGTA concentration. Similar to experiment [52], cells exhibit individual increases of 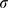 and 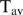 with a slope of the shift close to 1 comparable with the population slopes for the two measuring periods.

### Influence of buffer kinetics

BAPTA and EGTA are common 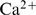 buffers to suppress 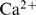 signals and we have used both in experiments [5]. Cells responded much more sensitive to BAPTA than to EGTA. BAPTA has much larger binding and dissociation rate constants than EGTA (Table 1). A disadvantage of the experiment is that the buffer is loaded into a cell by its esterificated form and the total amount that has entered is unknown and difficult to control. Here, we use modelling to illuminate the influence of the different buffer kinetics and concentrations of EGTA and BAPTA.

**Table 1:** Physiologic standard parameters used in simulation if not stated otherwise.

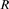	10 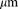	cell radius [62]
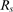	8 nm	channel radius [63]
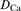	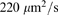	diffusion coefficient of cytosolic 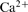 [64]
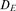	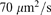	estimated diffusion coefficient of lumenal 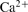 [65]
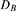	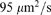	diffusion coefficient of mobile buffer [66]
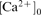	50 nM	standard 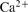 base level concentration [67]
[IP 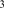 ]	0.1 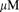	standard 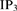 concentration [67]
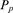	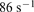	estimated pump rate constant [43]
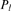	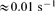	leak flux constant [68]
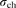	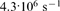	channel flux constant [43]
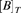	50 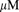	total mobile buffer concentration
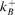	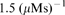	capture rate of EGTA [69]
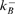	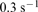	dissociation rate of EGTA [69]
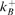	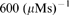	capture rate of BAPTA [70]
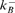	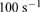	dissociation rate of BAPTA [70]
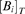	30 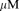	total immobile buffer concentration
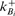	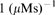	capture rate of the immobile buffer [70]
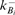	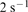	dissociation rate of the immobile buffer [70]

The definitions for the dissociation constants read 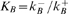 and 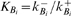.

**Table 2:** Definition of scaling factors and non-dimensional parameters.

Rescaling of time and space		
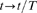		scaling time t with reaction time 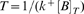
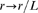		scaling space r with diffusion length 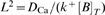
Dimensionless parameter definition		
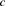	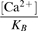	dimensionless free 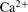 concentration
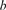	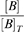	dimensionless free mobile buffer concentration
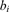	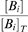	dimensionless free immobile buffer concentration
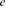	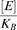	dimensionless free 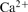 concentration within the ER
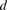	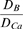	ratio of the diffusion coefficients
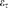	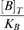	time separation of the mobile buffer
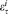	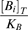	time separation of the immobile buffer
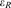	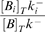	ratio of buffer influence
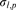	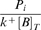	scaled fluxes of 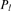 and 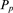
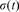	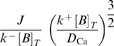	scaled channel cluster current 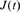
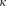	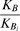	dissociation constants ratio of the mobile and immobile buffer

Figure 5D shows the dependence of 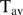 for fixed cell parameters on the buffer concentration in magenta for EGTA and in black for BAPTA, where squares denote simulations with a single channel current of 0.12 pA and the dots correspond to 1.2 pA. The larger current was achieved by an increased lumenal 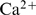 concentration. Cells only differ in the buffer type. We see that increasing BAPTA has a stronger effect than EGTA, which is mainly caused by the larger capture rate. Moreover, we observe a nonlinear dependence of 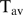 on the buffer concentration. The nonlinearity explains the individual shifts of cells in the 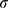
−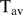 plane shown Figure 5D. The comparison of the two different current strengths for BAPTA (black) indicates the role of spatial coupling. Higher currents lead to stronger coupling, and subsequently increasing buffer concentrations have a smaller effect on 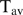.

### Cell characteristics in dependence on single channel currents

From the buffer simulations, we can determine the 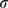
−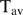 relation shown in Figure 5E. For the smaller currents, there is no qualitative difference between EGTA and BAPTA. Both exhibit a slope close to 1 as shown by the regression lines and an estimated deterministic time of 20 s. The simulations with higher cluster currents indicate a similar deterministic refractory period but the slope of the 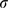
−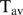 relation decreases to approximately 0.6. This might explain the experimentally found differences between cell types. Larger currents lead to stronger coupling on the macroscopic length scale and hence to smaller variations.

To confirm these findings and to test the dependency of the slope on other parameters, we analyze spiking of cells for the two different single channel currents. In each simulation set the cells have identical properties and differ only with respect to the buffer content leading to the distinct 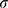 and 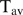 values in Figure 5E (see also Section 6 in Text S1). From these values we determine the population slopes 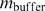. Figure 5F shows 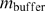 averaged over different spatial arrangements, 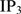 concentrations (stimulation levels) and pump strengths (see Figure 6 in Text S1). Analogously, we investigated 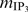, 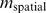 and 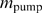 (data not shown). The results are very similar to those with 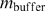. For smaller single channel current we obtain always a slope close to 1 when varying all 4 cell properties and for the larger current a slope to 0.6. Varying the buffer concentration, spatial arrangement of clusters, 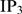 concentration or pump strength (within certain limits) does not change the 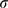
−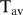 relation but only the position of the system on it.

## Discussion

We have presented here an efficient modelling concept for 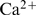 dynamics in 3 spatial dimensions. It simulates cell behavior starting from individual channels in full detail. Using Green's function and multiscale techniques allow for taking concentration gradients into account and thus for capturing the hierarchy of coupling strengths. The method can simulate up to 4000 seconds real time within 24 h on 8 CPUs for a cell with 32 clusters and 10 channels per cluster. In comparison to grid-based numerical methods, its main advantage is a gain of computational speed of several orders of magnitude, which enables us to simulate whole spike sequences. We demonstrate the potential of this modelling concept by simulating a variety of experiments. We compare the *in silico* data with time series obtained from spontaneous oscillations in cultured astrocytes, but several of the results will also apply to other cell types like those analyzed in [5].

These recent experiments showed for 4 different cell types that the sequences of interspike intervals in 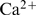 signalling are random [5]. In line with the ideas on the 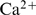 signalling mechanisms, we assumed single molecule state transitions to be a sufficient source of noise. We confirm this assumptions with our simulations here in which these state transitions are the only source of randomness. The fluctuations are carried up through the structural levels due to the existence of concentration gradients and hierarchies of coupling strength.

With our bottom-up modelling approach, we were able to generate all experimentally known 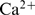 signal types in dependence on physiologic parameters. Spiking exhibits the random ISI sequences observed experimentally with fast regular sequences and slow irregular ones. In particular, the dependency on parameters of spatial coupling observed in experiments is reproduced. We find a sigmoidal response of the 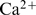 concentration upon very strong stimulation or strong spatial coupling, which is well known as over stimulation. We observe also bursting. We do not compare our bursting simulations with specific experiments here, but we would like to mention a general aspect. This signal type is usually ascribed to the existence of a dynamic feedback like store depletion or inhibition of 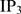 production which terminates bursts. Such a feedback is not required with a stochastic model. The random length of bursts in our stochastic model offers also a simple explanation for the irregular burst length observed in experiments.

With our method we are able to follow the 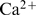 dynamics from the molecular to the cellular level. The single molecule fluctuations determine the global behavior, since they initiate cellular signals. Simultaneously, the local rough channel signal is smoothed on the cell level by the hierarchical system due to diffusion. The universality and variety of signalling cross talks between 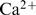 signalling and other pathways render 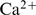 a potential source of noise in cellular systems. The fluctuations can be used for cell variability [4] with regards to gene regulation [53], [54] and cell differentiation [3] and provides a flexibility to changing external conditions which is needed during evolution [55].

### The 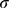
−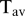 relation and functional robustness

Both the experiments and simulations show a simple linear relation between the standard deviation of ISI 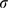 and the average ISI 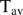. The existence of this linear relation turned out to be surprisingly robust. It survives even an increase of the single channel current by an order of magnitude. This relation describes for each individual cell the response to stimulation changes. Cells shift the spike pattern from slow and irregular to fast and regular along the 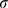
−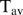 relation when we increase stimulation. That supplements the current ideas on frequency encoding [54], [56].

At the same time, the 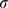
−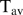 relation describes the outcome of spiking experiments with a group of cells. In the experiments, we subjected a sample of cells to the same protocol, and we obtained as many different responses as there are cells in the sample [5]. That set of responses is not arbitrarily scattered across the 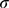
−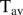 -plane but aligns along the 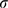
−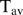 relation. All the variability among individual cells with respect to expression levels of pathway components, cell volume, ER volume, shape, ion concentration, etc. does not lead to severe deviations from this 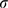
−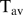 relation. 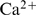 spiking is robust against variability of many pathway components in the sense that the 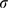
−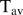 relation is robust. We learn from the simulations here, that it is rather the stochastic spike generation mechanism than control and regulation which provides for this robustness.

If we call the 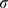
−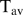 relation from a single cell obtained by parameter changes individual relation and that obtained from a sample of cells population relation, we can describe our findings as identity of individual and population relation.

We could reproduce the variability within a population of cells in simulations by varying cluster array geometry, pump strength, stimulation or buffering conditions. Changing these parameter values simply shifted the system on the 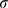
−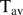 relation and did not modify the relation. But changing the single channel current by one order of magnitude did change the slope of the 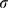
−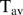 relation.

That suggests a mathematical definition of robustness which accounts for the fact that cells should be able to execute certain functions (e.g. to spike with a range of ISI), but not necessarily at the same strength of stimulation or normalized values of other parameters. We denote with 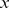 and 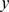 two variables describing the function (e.g. 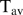 and 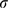), and with 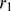,…,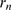 and 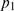,…,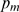 two sets of parameters (e.g. stimulation strength, temperature, cell volume). The relation between 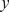 and 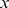 is robust with respect to value changes of parameters 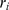, if it has the structure 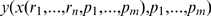. The parameters 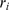 change only the value of 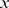 while the 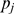 control also the properties of 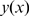, i.e. the properties of the pathway. We call this robustness of the function 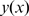 functional robustness (in difference to the robustness of the value of 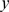). If we identify the stimulation strength with 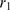, all cells distinguished by the values of 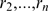 only can realize frequency encoding with the same 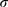
−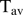 relation by varying 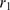. They can realize this function also by varying another 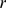-parameter or several of them: function and functional robustness are closely related.

The statement on robustness can also be interpreted with respect to identity of pathways converging onto 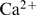 spiking. 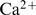 signals can be caused by many different stimuli. The pathways upstream from 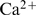 responding to the stimuli must differ with respect to their value of the 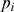, in order to be distinguishable by pathways downstream from 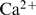.

In summary, cells realize frequency encoding - the function of 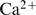 spiking - by mainly moving up and down the relation between standard deviation and average of ISI and to some degree by modulating the deterministic part of the ISI [52]. The 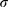
−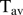 relation exists for a stochastic process only, since 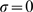 holds for deterministic systems. The 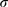
−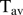 relation turned out to be functionally robust with respect to changes of values of one set of parameters. That set may describe cell variability within one cell type or pathway. Changing substantially another set of parameters modified the 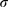
−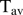 relation. That set appears rather to specify the identity of pathways converging on 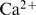 spiking.

### The role of IP_3_R clusters for astrocyte Ca^2+^ signalling

Our model predicts that close proximity of 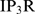 clusters is a prerequisite for a spontaneous 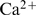 response to spread throughout a cell. Indeed, there are types of astrocytes in which 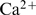 responses spread within the cell and those, such as Bergmann glia where this is not observed. Interestingly local, subcellular spontaneous 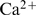 responses have been recorded which represent functional microdomains [57]. Complementary to the functional units, morphological units are described which are separated from each other by fine processes [58]. It is well conceivable that these thin processes separate endoplasmic reticulum between microdomains by more than 2 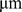 and according to our model this separation would prevent the spread of a local 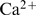 signal to other parts of the cell. In contrast, in cultured astrocytes, the endoplasmic reticulum is preferentially arranged around the cell center without apparent discontinuity [59] and these cells frequently exhibit spontaneous 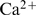 responses. In situ, spontaneous 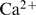 responses are reported for hippocampal astrocytes and these astrocytes are less polarized as compared to Bergmann glial cells and we would predict that they are less compartimentalized. Indeed, morphological studies indicate that hippocampal astrocytes have five to ten main processes from which smaller extensions branch off [60]. The synchronized activity obviously can spread within the volume of the main processes and soma of hippocampal astrocytes. Moreover, in contrast to culture, the endoplasmic reticulum in astrocytes in hippocampus tissue is preferentially located close to the plasma membrane [59]. These different morphological arrangements result in distinct patterns of 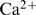 responses and as a consequence in different gene expression patterns [53].

### Do we need such a modelling tool beyond intracellular Ca^2+^ dynamics

The rise of cell imaging during the last decades illustrated the spatial structure of cells and protein localization. Obviously, cells are not homogeneous and active molecules coupled by diffusional transport are very common. Concentration gradients are functionally relevant, if they create microdomains inside which a pathway is in a state different from its state at other locations in the cell. They have been shown to exist for ‘the other’ fast diffusing intracellular messenger cAMP and in phosphorylation/dephosphorylation dynamics.

Hence, the need for spatially resolved cell models exists and we can apply the modelling concept, if all essential non-linearities are in the discrete active molecules or the boundary conditions and we can linearize remaining bulk reactions. The excellent validity of the linearization for the buffer reactions of 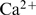 dynamics has been shown by Smith *et al.* [47]. We expect a degradation reaction like the cAMP degradation by PDEs also to be linearizable in good approximation. If local concentrations at active molecules should be outside the range of validity of the linearization, that can be fixed by the choice of the local quasi-static approximation of the diffusion process there in many cases. The non-linearities of cAMP production by membrane-bound adenylyl cyclase can be formulated as boundary condition and Green's function must then be used iteratively with an update of the boundary condition in each time step. These remarks illustrate that there is flexibility in the choice of reactions to be linearized which crucially expands the applicability of the concept.

## Methods

### Cell preparation

Astrocyte cell cultures were prepared from cortex of newborn NMRI mice [61]. Briefly, brain tissue was freed from blood vessels and meninges, trypsinised and gently triturated with a fire-polished pipette in the presence of 0.05% DNAase (Worthington Biochem. Corp., Freehold, NY, USA). Cells were washed twice and plated directly on poly-L-lysine (PLL; 100 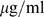 ; Sigma, Deisenhofen, Germany) coated glass coverslips (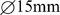) at densities of 3 to 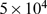 cells/coverslip, kept in 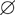-10-cm-dishes using Dulbecco's modified Eagle's medium (DMEM) supplemented with 10% fetal calf serum (FCS), 2 mM L-glutamine, 100 units/ml penicillin, and 100 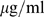 streptomycin. One day later, cultures were washed twice with Hank's balanced salt solution (HBSS).

Cells were maintained for at least 4 days and after reaching a subconfluent state, microglial cells and oligodendrocytes as well as their early precursors were dislodged by manual shaking and removed by washing with HBSS. The purity of the astrocytes was routinely determined by immunofluorescence using an antibody against glial fibrillary acidic protein (GFAP, Sigma), a specific astrocytic marker. The cultures typically exhibited more than 90% cells positive for GFAP.

### Cell imaging

Cultured cells plated on glass coverslips were measured between p4 and p6. Cells were incubated with the 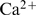 indicator dye Fluo-4-acetoxymethyl-ester (Fluo-4 AM, 5 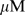, Molecular Probes, Eugene, USA) for 30 min at room temperature in HEPES buffer (148.9 mM NaCl, 5.4 mM KCl, 1 mM 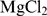, 10 mM 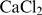, 10 mM HEPES, 5 mM D-glucose, pH 7.4) containing 0.01% Pluronic-127 (Molecular Probes). Subsequently cells were washed and kept in HEPES buffer for 15–20 min prior to the measurements with the conventional imaging system at a frequency of 0.33 Hz. Cultures were fixed within the microscope chamber of an upright microscope (Axioskop FS, Zeiss, Oberkochen, Germany) equipped with a 20× water immersion objective (UMPlanFl, numeric aperture: 0.5, Olympus, Hamburg, Germany) by a U-shaped platinum wire and superfused with HEPES buffer at 20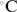. Substances were applied by changing the perfusate. Cells were illuminated (495 nm) from a monochromator (T.I.L.L. Photonics) and fluorescent images (515–545 nm) collected every 3 s with a 12 bit camera (SensiCam) on an upright microscope. At this state, no intercellular waves were observed. Single cell time series were extracted from these images with ImagingCellsEasily software.

## Supporting Information

Text S1 Detailed mathematical model and supporting results.
(0.75 MB PDF)

Video S1 The movie shows the free cytosolic calcium concentration during a spike lasting 15 s by an iso-concentration surface of 2 µM. The initial puff activates adjacent channel clusters by increasing their open probability. The clusters open and close randomly until inhibition terminates the release. Parameter values are in Table 1, the spatial arrangement of clusters is shown in Figure 1.
(9.94 MB AVI)
