# Supplementary material for: Calcium Signals Driven by Single Channel Noise
Source: PLoS Comput Biol. 2010 Aug 5;6(8):e1000870. doi: 10.1371/journal.pcbi.1000870 (PMC2917103; doi:10.1371/journal.pcbi.1000870)
Supplement: Text S1 — Detailed mathematical model and supporting results. (0.75 MB PDF) [file pcbi.1000870.s001.pdf]

# Supporting Text S1

# Calcium signals driven by single channel noise

Alexander Skupin, Helmut Kettenmann and Martin Falcke

## Contents

**1 $IP_3R$ model (Supporting Figure 1 and Table 1)** **3**

**2 Derivation of the linearized system (Supporting Figure 2 and Table 2)** **6**

**3 Deriving the solution (Supporting Figure 3)** **10**

3.1 $Ca^{2+}$ dynamics . . . . . . . . . . 14

3.2 Boundary conditions . . . . . . . . . . 17

3.3 Average concentrations . . . . . . . . . . 18

**4 Green's cell algorithm implementation** **20**

4.1 Gillespie algorithm . . . . . . . . . . 20

4.2 Parallel algorithm structure (Supporting Figure 4) . . . . . . . . . . 21

**5 Spiking in dependence on $IP_3$ and $Ca^{2+}$ (Supporting Figure 5)** **25**

**6 Population slopes (Supporting Figure 6)** **27**

## List of Figures


1 The DeYoung-Keizer model . . . . . . . . . . . . . . . . . . . . . . . . . . . 3

2 Linearization of the SERCA pump term . . . . . . . . . . . . . . . . . . . . . . 7

3 Sketch of the angles of two points in spherical coordinates . . . . . . . . . . . 14

4 Scheme of the parallel Green's cell model algorithm . . . . . . . . . . . . . . . 22

5 Channel dynamics in dependence on $IP_3$ and $Ca^{2+}$ . . . . . . . . . . . . . . . 26

6 Determination of population slope . . . . . . . . . . . . . . . . . . . . . . . 28


## List of Tables


1 Transition rates for the DeYoung-Keizer channel model . . . . . . . . . . . 4

2 Dimensionless parameter definition . . . . . . . . . . . . . . . . . . . . . . . . 8

# 1 $IP_3R$ model (Supporting Figure 1 and Table 1)

We use a modified version of the DeYoung-Keizer model (DKM), but the following derived reaction diffusion system and its solution can also be used with other channel models. The DKM assumes each $IP_3R$ to consist of four identical subunits with 3 binding sites each. One binding site for $IP_3$, one for $Ca^{2+}$ activating the subunit and another one for $Ca^{2+}$, which inhibits the subunit. The two binding sites for $Ca^{2+}$ with a higher affinity for the activating site and a lower affinity for the dominant inhibiting site is a minimal choice to generate nonlinearities which are essential for $Ca^{2+}$ induced $Ca^{2+}$ release.

Since each of the 3 binding sites can be free or occupied, a single subunit has $2^3$ different states $X_{ijk}$ and 12 possible transitions, which can be visualized on a cube as shown in Supporting Figure 1A.

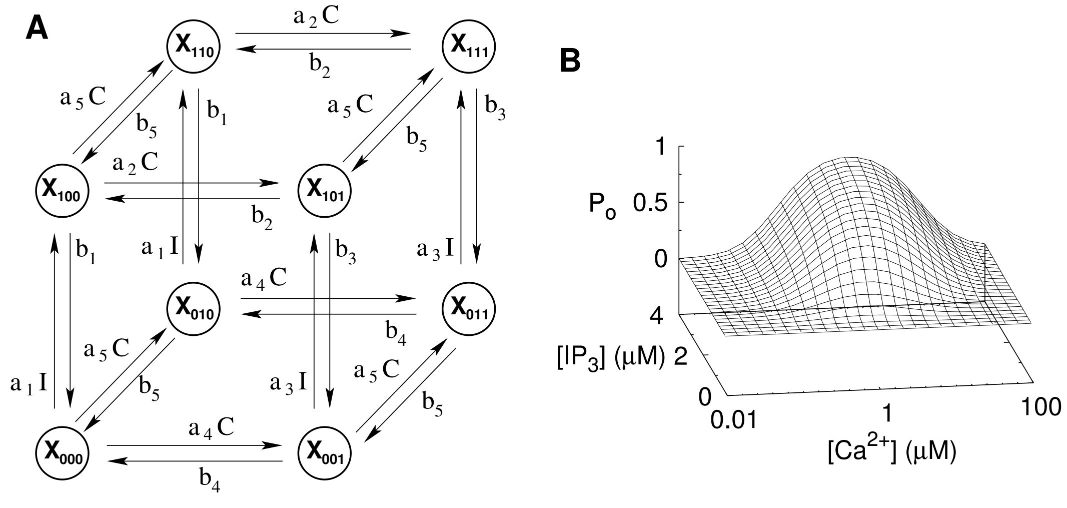


Supporting Figure 1: **A**: Scheme of the DeYoung-Keizer model for a single subunit. A subunit is active, if $IP_3$ (I) is bound and $Ca^{2+}$ (C) is only bound to the activating site, i.e. in state $X_{110}$. A channel opens if at least 3 of its 4 subunits are active. See text for more details and Supporting Table 1 for values of rates $b_i$ and rate constants $a_i$. **B**: Stationary open probability $P_o$ defined by Equation (2) for the rate constants $a_i$ and rates $b_i$ given in Supporting Table 1.

The first index of $X_{ijk}$ specifies $IP_3$ binding and is 1 if $IP_3$ is bound and 0 otherwise. Analogously, the second index indicates $Ca^{2+}$ binding to the activating site, and the last one corresponds to $Ca^{2+}$ binding to the dominant inhibiting site. A subunit is active in the

| | | |
|---|---|---|
| $a_1$ | 20 $(\mu Ms)^{-1}$ | $IP_3$ binding with no inhibiting $Ca^{2+}$ bound |
| $b_1$ | 20 $s^{-1}$ | $IP_3$ dissociation with no inhibiting $Ca^{2+}$ bound |
| $a_2$ | 0.001 $(\mu Ms)^{-1}$ | $Ca^{2+}$ binding to inhibiting site with $IP_3$ bound |
| $b_2$ | 0.03 $s^{-1}$ | $Ca^{2+}$ dissociation from inhib. site with $IP_3$ bound |
| $a_3$ | 2.6 $(\mu Ms)^{-1}$ | $IP_3$ binding with inhibiting $Ca^{2+}$ bound |
| $b_3$ | 20 $s^{-1}$ | $IP_3$ dissociation with inhibiting $Ca^{2+}$ bound |
| $a_4$ | 0.025 $(\mu Ms)^{-1}$ | $Ca^{2+}$ binding to inhib. site with no $IP_3$ bound |
| $b_4$ | 0.1 $s^{-1}$ | $Ca^{2+}$ dissociation from inhib. site without $IP_3$ |
| $a_5$ | 10 $(\mu Ms)^{-1}$ | $Ca^{2+}$ binding to activating site |
| $b_5$ | 1.225 $s^{-1}$ | $Ca^{2+}$ dissociation from activating site |

Supporting Table 1: Rates of the DKM used within simulations.

state $X_{110}$ only and a channel will open if at least three of the four subunits are activated.

The transitions between the states $X_{ijk}$ occur by stochastic binding and dissociation of signalling molecules to the corresponding binding sites. The rates for binding depend on the particular rate constants $a_i$ and on the $Ca^{2+}$ concentration $C$ and the $IP_3$ concentration $I$, respectively, as shown in Supporting Figure 1A, whereas dissociation occurs with constant rates $b_i$. Values are given in Supporting Table 1.

The binding and unbinding of $Ca^{2+}$ and $IP_3$ in an ensemble of receptors lead to a fraction of channels $p_{ijk}$ in the state $X_{ijk}$. In the case of a large and homogeneous ensemble, the dynamics of $p_{ijk}$ can be described by rate equations taking the dependence on the $Ca^{2+}$ and $IP_3$ concentration into account. In general these concentrations are not constant in time, and especially the $Ca^{2+}$ concentration changes enormously by transitions from closed to open states and vice versa. We can determine the stationary values $\bar{p}_{ijk}$ for constant $Ca^{2+}$ and $IP_3$ concentrations denoted by $C_{st}$ and $I$, respectively. They are given by

$$\bar{p}_{000} = \gamma_1\ d_1 d_2 d_5 \qquad \bar{p}_{100} = \gamma_1\ d_2 d_5\ I\ , \tag{1a}$$

$$\bar{p}_{010} = \gamma_1\ d_1 d_2\ C_{st} \qquad \bar{p}_{001} = \gamma_1\ d_3 d_5\ C_{st}\ , \tag{1b}$$

$$\bar{p}_{011} = \gamma_1\ d_3\ C_{st}^2 \qquad \bar{p}_{101} = \gamma_1\ d_5\ I\ C_{st}\ , \tag{1c}$$

$$\bar{p}_{110} = \gamma_1\ d_2\ I\ C_{st} \qquad \bar{p}_{111} = \gamma_1\ I\ C_{st}^2\ , \tag{1d}$$

where $\gamma_1^{-1} = (C_{st} + d5)(d_1 d_2 + C_{st} d_3 + C_{st} I + d_2 I)$ and $d_i = \mathrm{b_i}/\mathrm{a_i}$.

With these relations we can determine the stationary open probability $P_o$ in dependence on $Ca^{2+}$ and $IP_3$. Since a channel opens if three or four subunits are in the state $X_{110}$, the open probability takes the form

$$P_o = 4p_{110}^3 - 3p_{110}^4 \ , \tag{2}$$

which is shown in Supporting Figure 1B in dependence on $Ca^{2+}$ and $IP_3$ for the rates given in Supporting Table 1. We observe a bell shaped dependence on $Ca^{2+}$ and the monotonic increase of $P_o$ with increasing $IP_3$. The values of $P_o$ are in the range found experimentally [1, 2].

# 2 Derivation of the linearized system (Supporting Figure 2 and Table 2)

To reflect the most important cytosolic properties, we take free [$\mathrm{Ca}^{2+}$] one mobile [$B$] and one immobile buffer [$B_i$] in the cytosol into account leading to the following reaction diffusion system

$$\frac{\partial[\mathrm{Ca}^{2+}]}{\partial t} = D_{\mathrm{Ca}}\nabla^2[\mathrm{Ca}^{2+}] - P_p[\mathrm{Ca}^{2+}] + P_l([E]-[\mathrm{Ca}^{2+}]) + \sum_{j=1}^{N_{\mathrm{cl}}} J_j(t)\,\delta\left(\mathbf{r}-\mathbf{r_i}\right) \tag{3a}$$

$$- k^+[B][\mathrm{Ca}^{2+}] + k^-([B]_T-[B]) - k_i^+[B_i][\mathrm{Ca}^{2+}] + k_i^-([B_i]_T-[B_i])$$

$$\frac{\partial[B]}{\partial t} = D_B\nabla^2[B] - k^+[B][\mathrm{Ca}^{2+}] + k^-([B]_T-[B]) \tag{3b}$$

$$\frac{\partial[B_i]}{\partial t} = -k_i^+[B_i][\mathrm{Ca}^{2+}] + k_i^-([B_i]_T-[B_i]), \tag{3c}$$

where we applied the buffer conservation and used a linear pump and leak flux with the flux constants $P_p$ and $P_l$. $J_j(t)$ is the stochastically in time varying channel cluster current of the $j$th cluster, $k^+$ and $k^-$ denote the capture and dissociation rates of the buffers, and $[B]_T$ and $[B_i]_T$ are the total mobile and immobile buffer concentrations. The linearization of the nonlinear pump term $J^{\mathrm{nl}}_{\mathrm{pump}} = V^{\max}\frac{[\mathrm{Ca}^{2+}]^2}{[\mathrm{Ca}^{2+}]^2+K_D^2}$ of the Form $J^{\mathrm{lin}}_{\mathrm{pump}} = P_{\mathrm{p}}[\mathrm{Ca}^{2+}]$ is shown in Supporting Figure 2, where $K_D$ is the dissociation constant of the pump. The parameter $P_{\mathrm{p}} = \sqrt{3}V^{\max}/(4K_D)$ is determined by $J^{\mathrm{lin}}_{\mathrm{pump}} = J^{\mathrm{nl}}_{\mathrm{pump}}$ at the inflection point of $J^{\mathrm{nl}}_{\mathrm{pump}}$. The linearization matches the nonlinear pump term rather well for the most relevant range up to 2 $K_D$.

We introduce dimensionless concentrations by

$$c = \frac{[Ca^{2+}]}{K_B},\ b = \frac{[B]}{[B]_T},\ b_i = \frac{[B_i]}{[B_i]_T},\ e = \frac{[E]}{K_B} \tag{4}$$

where $K_B$ denotes the dissociation constant of the mobile buffer. For one channel cluster,

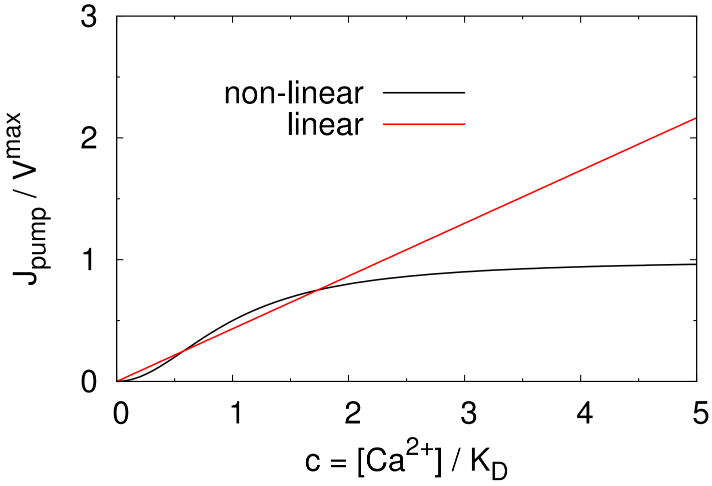


Supporting Figure 2: Linearization of the SERCA pump term. The linearization (red) of the nonlinear term $J^{\mathrm{nl}}_{\mathrm{pump}} = V^{\max}[\mathrm{Ca}^{2+}]^2 \left([\mathrm{Ca}^{2+}]^2 + K_D^2\right)^{-1}$ (black) around its inflection point leads to a quantitatively well approximation over a large concentration range. For regions, where the concentration is even higher than 2 $K_D$, i.e. close to open channels, the dynamics is mostly determined by the diffusion and channel terms.

this leads to

$$\begin{aligned}
\frac{1}{Tk^+[B]_T}\frac{\partial c}{\partial t} &= \frac{D_{Ca}}{L^2k^+[B]_T}\nabla^2 c - bc + (1-b) + \frac{P_l}{k^+[B]_T}(\bar{e}-c) - \frac{P_p}{k^+[B]_T}c \\
&\quad + \frac{[B_i]_T k_i^-}{[B]_T k^-}\left(1 - b_i - b_i c\frac{K_B}{K_{B_i}}\right) + \frac{J(t)}{L^3k^-[B]_T}\delta(\boldsymbol{r}-\boldsymbol{r}_0) && \text{(5a)}\\
\frac{1}{Tk^-}\frac{\partial b}{\partial t} &= \frac{D_B}{L^2k^-}\nabla^2 b - bc + (1-b) && \text{(5b)}\\
\frac{[B_i]_T}{T[B]_Tk^-}\frac{\partial b_i}{\partial t} &= \frac{[B_i]_T k_i^-}{[B]_T k^-}\left(1 - b_i - b_i c\frac{K_B}{K_{B_i}}\right)\ . && \text{(5c)}
\end{aligned}$$

To obtain a dimensionless system we choose

$$\begin{aligned}
\frac{D_{Ca}}{L^2k^+[B]_T} &= 1 \Rightarrow L^2 = \frac{D_{Ca}}{k^+[B]_T} && \text{(6a)}\\
\frac{1}{Tk^+[B]_T} &= 1 \Rightarrow T = \frac{1}{k^+[B]_T} && \text{(6b)}
\end{aligned}$$

defining the diffusion length $L$ and reaction time $T$. They are used to rescale time $t \to t/T$ and space $r \to r/L$. Similar the remaining quantities in equations (5) can be subsumed

| | | |
|---|---|---|
| $c$ | $\frac{[\mathrm{Ca}^{2+}]}{K_B}$ | dimensionless free $Ca^{2+}$ concentration |
| $b$ | $\frac{[B]}{[B]_T}$ | dimensionless free mobile buffer concentration |
| $b_i$ | $\frac{[B_i]}{[B_i]_T}$ | dimensionless free immobile buffer concentration |
| $e$ | $\frac{[E]}{K_B}$ | dimensionless free $Ca^{2+}$ concentration within the ER |
| $d$ | $\frac{D_B}{D_{Ca}}$ | ratio of the diffusion coefficients |
| $\epsilon_\tau$ | $\frac{[B]_T}{K_B}$ | time separation of the mobile buffer |
| $\epsilon_\tau^i$ | $\frac{[B_i]_T}{K_B}$ | time separation of the immobile buffer |
| $\epsilon_R$ | $\frac{[B_i]_T k_i^-}{[B]_T k^-}$ | ratio of buffer influence |
| $\sigma_{\mathrm{l,p}}$ | $\frac{\sigma_i}{k^+[B]_T}$ | scaled fluxes of $P_l$ and $P_p$ |
| $\sigma(t)$ | $\frac{J}{k^-[B]_T}\left(\frac{k^+[B]_T}{D_{Ca}}\right)^{\frac{3}{2}}$ | scaled channel cluster current $J(t)$ |
| $\kappa$ | $\frac{K_B}{K_{B_i}}$ | dissociation constants ratio of the mobile and immobile buffer |

Supporting Table 2: Definition of non-dimensional parameters as given in Table 1 in the main text.

in dimensionless parameters given in Supporting Table 2 and in Table 2 of the main text.

Thus equations (5) take the form

$$\frac{\partial c}{\partial t} = \nabla^2 c - (bc + b - 1) - \epsilon_R (b_i c\kappa + b_i - 1) - \sigma_p c + \sigma_l(\bar{e} - c) + \sum_{i=1}^{N_{\mathrm{cl}}} \sigma(t)\delta(\boldsymbol{r} - \boldsymbol{r}_i) \tag{7a}$$

$$\epsilon_\tau \frac{\partial b}{\partial t} = d_\epsilon \nabla^2 b - (bc + b - 1) \tag{7b}$$

$$\epsilon_\tau^i \frac{\partial b_i}{\partial t} = -\epsilon_R (b_i c\kappa + b_i - 1) \ . \tag{7c}$$

In order to solve these equations analytically we linearize them around the initial state which is assumed to be the stationary state when all channels are closed. The scaled initial conditions read

$$c_0 = \frac{[Ca^{2+}]_0}{K_B}, \quad b_0 = \frac{1}{c_0 + 1}, \quad b_{i,0} = \frac{1}{c_0\kappa + 1}, \tag{8a}$$

where $\kappa = \frac{K_B}{K_{B_i}}$ denotes the ratio of the dissociation constants of the mobile and immobile buffer respectively. Replacing $c = c_0 + \delta c$, $\bar{e} = \bar{e}_0 + \delta\bar{e}$, $b = b_0 + \delta b$ and $b_i = b_{i,0} + \delta b_i$ in

equations (7) we get

$$\frac{\partial \delta c}{\partial t} = \nabla^2 \delta c - [(1+c_0)\delta b + \epsilon_R(1+\kappa c_0)\delta b_i + (b_0 + b_{i,0}\epsilon_R\kappa + \sigma_p - \sigma_l)\delta c + \delta c \delta b + \epsilon_R \kappa \delta c \delta b_i] + \sigma_l \delta \bar{e} + \sum_{i=1}^{N_{\rm cl}} \sigma(t)\delta(\boldsymbol{r} - \boldsymbol{r}_i) \quad (9a)$$

$$\epsilon_\tau \frac{\partial \delta b}{\partial t} = d_\epsilon \nabla \delta b - [(1+c_0)\delta b + b_0 \delta c] - \delta c \delta b \quad (9b)$$

$$\epsilon_\tau^i \frac{\partial b_i}{\partial t} = -\epsilon_R \left[(1+\kappa c_0)\delta b_i + b_{i,0}\kappa \delta c\right] - \epsilon_R \kappa \delta c \delta b_i \ . \quad (9c)$$

We now neglect all nonlinear terms in (9) in $\delta c$, $\delta b$ and $\delta b_i$ and end up with the linearized dimensionless system of the form

$$\frac{\partial \delta c}{\partial t} = \nabla^2 \delta c - [(1+c_0)\delta b + \epsilon_R(1+\kappa c_0)\delta b_i + (b_0 + b_{i,0}\epsilon_R\kappa + \sigma_p - \sigma_l)\delta c] + \sigma_l \delta \bar{e} + \sum_{i=1}^{N_{\rm cl}} \sigma(t)\delta(\boldsymbol{r} - \boldsymbol{r}_i) \quad (10a)$$

$$\epsilon_\tau \frac{\partial \delta b}{\partial t} = d_\epsilon \nabla^2 \delta b - [(1+c_0)\delta b + b_0 \delta c] \quad (10b)$$

$$\epsilon_\tau^i \frac{\partial b_i}{\partial t} = -\epsilon_R \left[(1+\kappa c_0)\delta b_i + b_{i,0}\kappa \delta c\right] \ . \quad (10c)$$

For more convenient reading we drop the $\delta$s of the concentrations and find the system

$$\frac{\partial c}{\partial t} = \nabla^2 c - [(1+c_0)b + \epsilon_R(1+\kappa c_0)b_i + (b_0 + b_{i,0}\epsilon_R\kappa + \sigma_p - \sigma_l)c] + \sigma_l \bar{e} + \sum_{i=1}^{N_{\rm cl}} \sigma(t)\delta(\boldsymbol{r} - \boldsymbol{r}_i) \quad (11a)$$

$$\frac{\partial b}{\partial t} = d\,\nabla^2 b - \frac{1}{\epsilon_\tau}\left[(1+c_0)b + b_0 c\right] \quad (11b)$$

$$\frac{\partial b_i}{\partial t} = -\frac{\epsilon_R}{\epsilon_\tau^i}\left[(1+\kappa c_0)b_i + b_{i,0}\kappa c\right] \quad (11c)$$

given in the main text with the definitions of $\sigma_{\rm m} = (1+c_0)$, $\sigma_{\rm im} = \epsilon_R(1+\kappa c_0)$ and $\sigma_{\rm c} = (b_0 + b_{i,0}\epsilon_R\kappa + \sigma_{\rm p} - \sigma_{\rm l})$.

# 3 Deriving the solution (Supporting Figure 3)

The model uses Green's functions [3, 4] to determine the concentrations. The solution of a partial differential equation calculated by Green's function is

$$\begin{aligned} C(\mathbf{r},t) &= \int_V d\mathbf{r}' F_0(\mathbf{r}') G(\mathbf{r},t|\mathbf{r}',\tau) + \int_S dS' \int_0^t d\tau \Phi(\mathbf{r}',\tau) G(\mathbf{r},t|\mathbf{r}',\tau) \\ &+ \int_V d\mathbf{r}' \int_0^t d\tau F(\mathbf{r}',\tau) G(\mathbf{r},t|\mathbf{r}',\tau) \ , \end{aligned} \tag{12}$$

where $V$ denotes the cell volume and $S$ the cell surface. The solution depends on the initial concentration distribution $F_0(\mathbf{r}')$, the time dependent boundary condition $\Phi(\mathbf{r}',\tau)$ and the volume production term $F(\mathbf{r}',\tau)$.

Before we solve the system (11) by coupled Green's functions for a spherical cell with radius $R$, we first determine the Green's function for a single component system, i.e. neglect buffer reactions and coupling with the ER.

The general equation in spherical coordinates reads

$$\frac{\partial c}{\partial t} = D\nabla^2 c + g(r,\theta,t) \tag{13a}$$

$$c(r,\theta,0) = f(r,\theta) \tag{13b}$$

$$\left.\frac{\partial c}{\partial r}\right|_{r=R} = j(\theta,t) \tag{13c}$$

$$\left.c\right|_{r=R} = C_R \tag{13d}$$

where $g(r,\theta,t)$ is a source density depending on $r$ and $\theta$ and $t$ and $f(r,\theta)$ denotes the initial condition. The boundary conditions given by $j(\theta,t)$ for no-flux conditions (13c) specifies influx through the cell membrane or is given in case of Dirichlet boundary conditions (13d) by the concentration at the surface.

The Green's function is the response of a system at point $P(\mathbf{r})$ at time $t$ due to a $\delta$ source in time ($t'$) and space at point $P'(\mathbf{r}')$. Hence, for two points we can use the symmetry and neglect first the $\phi$ dependence which can be incorporated later by trigonometric properties.

The corresponding equation of Equation (13) for the Green's function $G = G(r, \theta, t|r', \theta', t')$ takes the form

$$\begin{aligned} \frac{\partial G}{\partial t} &= D\nabla^2 G + \frac{1}{r'^2 \sin\theta'}\delta(r-r')\delta(\theta-\theta')\delta(t-t') && \text{(14a)} \\ G(r,\theta,t|r',\theta',t') &= 0, \quad t \geq t' \,, && \text{(14b)} \end{aligned}$$

where $G$ has to fulfil the corresponding boundary condition in (13). This problem can be solved by Laplace transformation and separation ansatz. After Laplace transform with respect to $t$ the governing equation of the transformed Green's function $\tilde{G}(r, \theta, s|r', \theta', t')$ reads

$$\tilde{G} = D\nabla^2\tilde{G} + \frac{1}{r'^2 \sin\theta'}\delta(r-r')\delta(\theta-\theta')e^{-st'} \,. \tag{15}$$

We first solve the homogeneous problem being the Helmholtz equation

$$\nabla^2\psi(r,\theta) + \lambda^2\psi(r,\theta) = 0 \tag{16}$$

where the $\lambda$s are determined by the particular boundary condition.

It reads in spherical coordinates

$$\frac{\partial^2\psi}{\partial r^2} + \frac{2}{r}\frac{\partial\psi}{\partial r} + \frac{1}{r^2\sin\theta}\frac{\partial}{\partial\theta}\left[\sin\theta\frac{\partial\psi}{\partial\theta}\right] = -\lambda^2\psi \,, \tag{17}$$

which can be solved by a standard separation ansatz. We expand the space dependent part $D\nabla^2\tilde{G}$ in eigenfunctions of the Laplace operator $\nabla^2$. The radial part leads to Bessel's differential equation and the angle dependent part obeys a Legendre differential equations. Due to convergence restrictions, the solution of the Helmholtz equation (16) takes the form

$$\begin{aligned} \psi_{lp}(r,\theta) &= \frac{J_{l+1/2}(\lambda_{lp}r)}{r^{1/2}}P_l(\cos\theta), \quad p = 1,2,3,\ldots \; l = 0,1,2,\ldots && \text{(18)} \\ \psi_{00}(r,\theta) &= 1 \,, && \text{(19)} \end{aligned}$$

where $J_{l+1/2}(x)$ denotes the Bessel function of the first kind, $P_l(\cos\theta)$ is the Legendre

polynomial and $\lambda_{lp}$ is determined for no-flux boundary condition by

$$\frac{\partial}{\partial r}\frac{J_{l+1/2}(\lambda_{lp}r)}{r^{1/2}}\bigg|_{r=R} = \frac{l}{R\lambda_{lp}}J_{l+1/2}(\lambda_{lp}R) - J_{l+3/2}(\lambda_{lp}R) = 0. \tag{20}$$

Thus we can solve Equation (15) by inserting the ansatz

$$\tilde{G}(r,\theta,s|r',\theta',t') = \sum_{l,p=0}^{\infty}\beta_{l,p}\psi_{l,p}(r,\theta). \tag{21}$$

leading to

$$s\sum_{l,p=0}^{\infty}\beta_{l,p}\psi_{l,p}(r,\theta) = -D\sum_{l,p=0}^{\infty}\beta_{l,p}\lambda_{lp}^2\psi_{l,p}(r,\theta) + \frac{1}{r'^2\sin\theta'}\delta(r-r')\delta(\theta-\theta')e^{-st'} . \tag{22}$$

By applying the integral-operators

$$\int_{-1}^{+1} d\mu\, P_m(\mu) \tag{23a}$$

$$\int_0^b dr\, r^{3/2}J_{m+1/2}(\lambda_{mq}r) \tag{23b}$$

we get due to orthogonality

$$s\beta_{m,q} = -\beta_{m,q}\lambda_{mq}^2 D + \frac{1}{\mathcal{N}(m)\mathcal{N}(\lambda_{mq})}\psi_{mq}(r',\theta')e^{-st'} , \tag{24}$$

where the norms $\mathcal{N}$ are given by

$$\mathcal{N}(l) = \int_{-1}^{+1} d\mu\, P_l^2(\mu) = \frac{2}{2l+1} \tag{25a}$$

$$\mathcal{N}(\lambda_{lp}) = \int_0^R dr\, r^2\left[\frac{J_{l+1/2}(\lambda r)}{r^{1/2}}\right]^2 \tag{25b}$$

$$= \frac{R^2}{2}\left[J_{l+1/2}^2(\lambda_{lp}R) - J_{l-1/2}(\lambda_{lp}R)J_{l+3/2}(\lambda_{lp}R)\right]$$

$$\mathcal{N}(\lambda_{00}) = \int_{-1}^{+1} d\mu\int_0^R dr\, r^2 = 2\frac{R^3}{3}. \tag{25c}$$

Equation (24) determines the unknown coefficients $\beta_{lp}$. The solution in Laplace space is

thus given by

$$\tilde{G}(r,\theta,s|r',\theta',t') = \sum_{l,p=0}^{\infty} \frac{1}{\mathcal{N}(l)\mathcal{N}(\lambda_{lp})(s+D\lambda_{lp}^2)} \psi_{lp}(r',\theta') e^{-st'} \psi_{l,p}(r,\theta). \tag{26}$$

It can be transformed back easily to time by the residual theorem, since we have first order poles, $s + D\lambda^2$, along the negative real axis only. The Green's function of the inhomogeneous diffusion problem (13) without the $\phi$ dependence finally is

$$\begin{aligned} G(r,\theta,t|r',\theta',t') &= \sum_{l=0,p=1}^{\infty} \frac{1}{\mathcal{N}(l)\mathcal{N}(\lambda_{lp})} \frac{J_{l+1/2}(\lambda_{lp} r')}{r'^{1/2}} P_l(\cos\theta') e^{\lambda_{lp}^2 D t'} \\ & \qquad \frac{J_{l+1/2}(\lambda_{lp} r)}{r^{1/2}} P_l(\cos\theta) e^{-\lambda_{lp}^2 D t} + \frac{3}{2R^3} \ . \end{aligned} \tag{27}$$

For simulation of a cell the spherical symmetry is not valid and we have to introduce explicitly the $\phi$ dependence in the Green's function (27). This only depends on the cosines of the angles of the two points $P(\mathbf{r})$ and $P'(\mathbf{r}')$, and hence we can rotate the coordinate system such as one of the angles is zero leading to $P_l(\cos\theta) = 1$. The angle $\Theta$ between the points is given by

$$\cos(\Theta) = \cos(\theta)\cos(\theta') + \sin(\theta)\sin(\theta')\cos(\phi - \phi') \ , \tag{28}$$

as shown Supporting Figure 3.

The Green's function takes the form

$$\begin{aligned} G(r,\theta,\phi,t|r',\theta',\phi',t') &= \sum_{l=0,p=1}^{\infty} \frac{J_{l+1/2}(\lambda_{lp} r) J_{l+1/2}(\lambda_{lp} r')}{2\pi \mathcal{N}(l)\mathcal{N}(\lambda_{lp})\sqrt{rr'}} P_l(\cos\Theta) \times \\ & \qquad e^{-\lambda_{lp}^2 D(t-t')} + \frac{3}{4\pi R^3} \ , \end{aligned} \tag{29}$$

where the $\phi$ dependence gives another normalization factor of $1/(2\pi)$. $D$ denotes the diffusion coefficient and the norms $\mathcal{N}$ are given by (25). The $\lambda_{lp}$s are determined by the corresponding boundary conditions as described in Section 3.2.

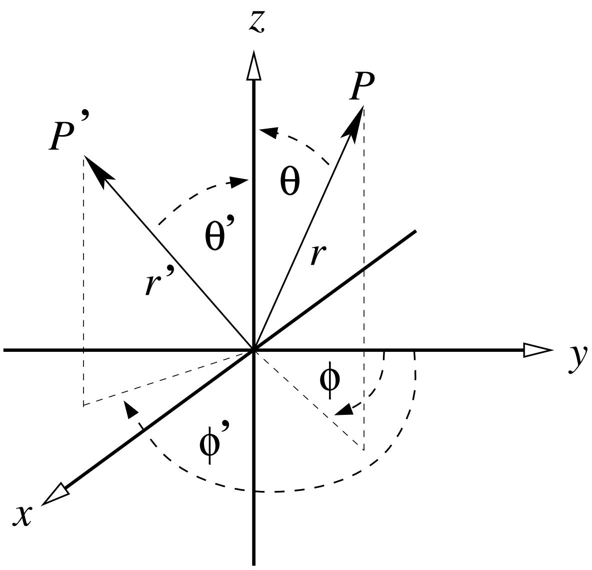


Supporting Figure 3: Sketch of the angles of two points $P$ and $P'$ in spherical coordinates. For more than two asymmetrically distributed points we have to incorporate the $\phi$ dependence. Since the solution (27) does only depend on the cosine of the angle between the two points $P$ and $P'$, we can use Equation (28) to rotate the system with respect to the coordinates of the two points.

### 3.1 $Ca^{2+}$ dynamics

We now introduce the coupling with buffer reactions. Therefore we write the RDS (11) in matrix form

$$\left[\begin{pmatrix} 1 & 0 & 0 \\ 0 & d & 0 \\ 0 & 0 & 0 \end{pmatrix} \nabla^2 - \begin{pmatrix} 1 & 0 & 0 \\ 0 & \epsilon_\tau & 0 \\ 0 & 0 & \epsilon_\tau^i \end{pmatrix} \frac{\partial}{\partial t} + \begin{pmatrix} a_{11} & a_{12} & a_{13} \\ a_{21} & a_{22} & 0 \\ a_{31} & 0 & a_{33} \end{pmatrix}\right] \begin{pmatrix} c \\ b \\ b_i \end{pmatrix} = - \begin{pmatrix} f_c(\mathbf{r}, t) \\ f_{b_m}(\mathbf{r}, t) \\ f_{b_i}(\mathbf{r}, t) \end{pmatrix} . \quad (30)$$

In order to solve this system of coupled PDEs by coupled Green's functions or a Green's dyadic $\mathbf{G}$ [5], we have to solve similar to Equation (14) the following problem,

$$\mathcal{L}\mathbf{G} = \begin{pmatrix} \nabla^2 - \frac{\partial}{\partial t} + a_{11} & a_{12} & a_{13} \\ a_{21} & d_\epsilon \nabla^2 - \epsilon_\tau \frac{\partial}{\partial t} + a_{22} & 0 \\ a_{31} & 0 & -\epsilon_\tau^i \frac{\partial}{\partial t} + a_{33} \end{pmatrix} \begin{pmatrix} g_{11} & g_{12} & g_{13} \\ g_{21} & g_{22} & g_{23} \\ g_{31} & g_{32} & g_{33} \end{pmatrix}$$

$$= -\frac{1}{r'^2 \sin\theta'} \delta(r - r')\delta(\theta - \theta')\delta(t - t') \begin{pmatrix} 1 & 0 & 0 \\ 0 & 1 & 0 \\ 0 & 0 & 1 \end{pmatrix} . \quad (31)$$

Analogously to (15) the time derivative can be replaced by $s$ due to a Laplace transform leading to

$$\tilde{\mathcal{L}}\tilde{\mathbf{G}} = -\frac{1}{r'^2 \sin\theta'} \delta(r - r')\delta(\theta - \theta') e^{-st'} \mathbf{1}_{3\times 3} . \quad (32)$$

With the same boundary conditions for calcium and the buffers, the system (30) can be solved by the spectral ansatz

$$\tilde{\mathbf{G}} = \sum_{l=0,p=0}^{\infty} \boldsymbol{\alpha}_{lp} \psi_{lp}(r, \theta), \quad (33)$$

where $\psi_{lp}(r, \theta)$ is the solution for the Helmholtz equation (18), which respects the appropriate boundary conditions. Thus, the Green's matrix is determined by the amplitude matrix $\boldsymbol{\alpha}_{lp}$. By inserting (33) into equation (32) we get

$$\sum_{l=0,p=0}^{\infty} \mathbf{M}_{lp} \;\; \boldsymbol{\alpha}_{lp} \;\; \psi_{lp} = -\frac{1}{r'^2 \sin\theta'} \delta(r - r')\delta(\theta - \theta') e^{-st'} \; \mathbf{1}_{3\times 3} . \quad (34)$$

By applying the integral operators (23) on both sides, the amplitude matrix is given by

$$\boldsymbol{\alpha}_{mq} = \frac{\psi_{mq}(r', \theta') e^{-st'}}{\mathcal{N}(m)\mathcal{N}(\lambda_{mq})} \, \mathbf{M}_{lp}^{-1} , \quad (35)$$

with the coupling matrix

$$\mathbf{M}_{lp} = \begin{pmatrix} \lambda_{lp}^2 + s\sigma_\mathrm{c} & \sigma_\mathrm{m} & \sigma_\mathrm{im} \\ b_0 & d\epsilon_\tau \lambda_{lp}^2 + s\epsilon_\tau + \sigma_\mathrm{m} & 0 \\ b_{i,0}\epsilon_r \kappa & 0 & s\epsilon_\tau^i + \sigma_\mathrm{im} \end{pmatrix} , \tag{36}$$

with the previously introduced shortcuts $\sigma_\mathrm{m} = (1 + c_0)$, $\sigma_\mathrm{im} = \epsilon_R(1 + \kappa c_0)$ and $\sigma_\mathrm{c} = (b_0 + b_{i,0}\epsilon_R\kappa + \sigma_\mathrm{p} - \sigma_\mathrm{l})$. Equation (34) can be transformed back into real space by the property of the matrix inversion

$$\mathbf{M}^{-1} = \frac{1}{|\mathbf{M}|}\mathrm{adj}(\mathbf{M}) , \tag{37}$$

which enables us to apply the residual theorem by determining the zeros of $|\mathbf{M}|$ leading to a cubic equation for $s$. Thus the Green's matrix takes the form

$$\tilde{\mathbf{G}}(r,\theta,t|r',\theta',t') = \sum_{l=0,p=1}^{\infty}\sum_{i=1}^{3} \frac{\mathrm{adj}(\mathbf{M}_{lp})}{\partial|\mathbf{M}_{lp}|/\partial s|_{s=s_i^{lp}}} \frac{1}{\mathcal{N}(l)\mathcal{N}(\lambda_{lp})} \frac{J_{l+1/2}(\lambda_{lp}r')}{r'^{1/2}} P_l(\cos\theta') e^{-s_i t'} \frac{J_{l+1/2}(\lambda_{lp}r)}{r^{1/2}} P_l(\cos\theta) e^{s_i t} + \frac{3}{2b_c^3} e^{s_i(t-t')} \frac{\mathrm{adj}(\mathbf{M}_{00})}{\partial|\mathbf{M}_{00}|/\partial s|_{s=s_i^{lp}}} \tag{38}$$

with the dimensionless cell radius $b_c$. If we now assume a $\delta$ source density describing a channel cluster

$$\sigma(t)\frac{\delta(r-r_c)\delta(\theta)}{r^2\sin\theta}\begin{pmatrix}1\\0\\0\end{pmatrix} \tag{39}$$

the concentrations at point $\mathbf{r}$ due to release at point $\mathbf{r}_c$ and uptake are given by

$$\begin{pmatrix}c\\b\\b_i\end{pmatrix}(\mathbf{r},t) = \sum_{l=0,p=1}^{\infty} \frac{J_{l+1/2}(\lambda_{lp}r)}{r^{1/2}} P_l(\cos\Theta) \begin{pmatrix}\chi_1^{(lp)}\\ \chi_2^{(lp)}\\ \chi_3^{(lp)}\end{pmatrix}(r_c,t) + \begin{pmatrix}\chi_1^{(00)}\\ \chi_2^{(00)}\\ \chi_3^{(00)}\end{pmatrix}(t) + \begin{pmatrix}c_\mathrm{out}\\ b_\mathrm{st}(c_\mathrm{out})\\ b_\mathrm{i,st}(c_\mathrm{out})\end{pmatrix} , \tag{40}$$

with the relation (28) for $P_l(\cos\Theta)$ (See Supporting Figure 3). The time and boundary dependent response functions read

$$\begin{pmatrix}\chi_1^{(lp)}\\ \chi_2^{(lp)}\\ \chi_3^{(lp)}\end{pmatrix}(r_c,t)=\sum_{i=1}^{3}\frac{1}{2\pi\mathcal{N}(l)\mathcal{N}(\lambda_{lp})}\frac{J_{l+1/2}(\lambda_{lp}r_c)}{r_c^{1/2}}\times \tag{41}$$

$$\int_0^t dt'\sigma(t')e^{s_i(t-t')}\frac{\mathrm{adj}(\mathbf{M}_{lp})}{\partial|\mathbf{M}_{lp}|/\partial s|_{s=s_i^{lp}}}\begin{pmatrix}1\\0\\0\end{pmatrix}$$

$$\begin{pmatrix}\chi_1^{(00)}\\ \chi_2^{(00)}\\ \chi_3^{(00)}\end{pmatrix}(t)=\sum_{i=1}^{3}\frac{3}{4\pi b_c^3}\int_0^t dt'\sigma(t')e^{s_i(t-t')}\frac{\mathrm{adj}(\mathbf{M}_{00})}{\partial|\mathbf{M}_{00}|/\partial s|_{s=s_i^{lp}}}\begin{pmatrix}1\\0\\0\end{pmatrix}, \tag{42}$$

taking into account the in time varying cluster current $\sigma(t)$. The last term describes possible extracellular induced concentrations $c_{\mathrm{out}}$, $b_{\mathrm{st}}$ and $b_{\mathrm{i,st}}$.

### 3.2 Boundary conditions

Intracellular $Ca^{2+}$ dynamics can be determined by Neumann (no-flux) boundary condition or by Dirichlet boundary condition in dependence on the cell type and kind of experiment. The boundary conditions at the plasma membrane $r = b_c$ (the scaled cell radius $R$) are reflected by the $\lambda_{lp}$s and the two last terms in Equation (40). No-flux boundary conditions require

$$\frac{\partial}{\partial r}\frac{J_{l+1/2}(\lambda_{lp}r)}{r^{1/2}}\bigg|_{r=b_c}=\frac{l}{b_c\lambda_{lp}}J_{l+1/2}(\lambda_{lp}b_c)-J_{l+3/2}(\lambda_{lp}b_c)=0. \tag{43}$$

and $c_{\mathrm{out}} = b_{\mathrm{st}}(c_{\mathrm{out}}) = b_{\mathrm{i,st}}(c_{\mathrm{out}}) = 0$ holds. Dirichlet boundary conditions for $c$ with $c(b_c) = c_{\mathrm{out}}$ require

$$J_{l+1/2}(\lambda_{lp}b_c) = 0. \tag{44}$$

$b_{\mathrm{st}}(c_{\mathrm{out}})$ and $b_{\mathrm{i,st}}(c_{\mathrm{out}})$ are the stationary values of the concentration dynamics with all channels closed. $\boldsymbol{\chi}^{(00)}$ vanishes with Dirichlet conditions.

The simulations in the main text were all done with Neumann boundary conditions since we focused on the influence of single channel behavior on the intracellular dynamics.

### 3.3 Average concentrations

The channel currents are set by the difference of the average $Ca^{2+}$ concentrations in the cytosol and in the ER (see Equation 1 in main paper). These concentrations can be determined by spatial integration over the whole cell.

If we do not assume $Ca^{2+}$ entry through the cell membrane, the total amount of $Ca^{2+}$ will stay constant

$$N_{\mathrm{tot}} = \left[(\bar{c}(t) - c_0) + (b_0 - \bar{b}(t)) + (b_{i,0} - \bar{b_i}(t)) + \bar{e}(t)/\gamma\right] V_{\mathrm{cyt}} = const\,. \tag{45}$$

With the assumption that at time $t = 0$ the cell has no open channels and is in equilibrium we have also the relation

$$N_{\mathrm{tot}} = (c_0 + (b_T - b_0) + (b_{i,T} - b_{i,0}) + \bar{e}_0/\gamma)V_{\mathrm{cyt}}\,, \tag{46}$$

where $\gamma$ is the volume ratio between the cytosol and the ER. Hence, to calculate the average $Ca^{2+}$ concentration within the ER $\bar{e}(t)$, we rely on the average concentrations $\bar{\mathbf{c}}$ of all three components. Since $V_{\mathrm{cyt}}\bar{\mathbf{c}} = \int_{V_{\mathrm{cell}}} dV\mathbf{c}$ we have to integrate the solution (40) over the entire cell:

$$\bar{\mathbf{c}} = \frac{1}{V_{\mathrm{cyt}}} \int_0^{b_c} \int_0^{\pi} \int_0^{2\pi} \boldsymbol{c}\, r^2 \sin(\theta)\, dr\, d\theta\, d\phi\,. \tag{47}$$

The $\phi$ integration simply gives a factor of $2\pi$ whereas the other two integrations lead to

the equations

$$\mathcal{R} = \int_0^{b_c} \frac{J_{l+1/2}(\lambda_{lp} r)}{r^{1/2}} r^2 dr$$

$$= \frac{2^{-\left(\frac{3}{2}+l\right)}\, b_c^{(3+l)}\, \lambda_{lp}^{\frac{1}{2}+l}\, \Gamma\left(\frac{3+l}{2}\right)}{\Gamma\left(\frac{5+l}{2}\right)\Gamma\left(\frac{3}{2}+l\right)}\, {}_1F_2\left[\frac{3+l}{2}; \left(\frac{5+l}{2}, \frac{3+l}{2}\right); -\left(\frac{b_c\lambda_{lp}}{2}\right)^2\right] \tag{48a}$$

$$\mathcal{Q} = \int_0^{\pi} P_l(\cos\theta)\sin\theta d\theta = \int_{-1}^{1} P_l(x)dx = \frac{2\sin(l\pi)}{l\pi + l^2\pi} = \begin{cases} 0 & , \quad l > 0 \\ 2 & , \quad l = 0 \end{cases} , \tag{48b}$$

where ${}_1F_2\left[x, \mathbf{y}, z\right]$ denotes the hyper-geometric function [6]. Hence, only modes with $l = 0$ contribute to the global concentration and Equation (48a) can be simplified to

$$\mathcal{R} = \sqrt{\frac{2}{\pi}} \frac{\sin\left(\lambda_{0p} b_c\right) - b_c \lambda_{0p} \cos\left(\lambda_{0p} b_c\right)}{\lambda_{0p}^{5/2}} . \tag{49}$$

With this solution we can calculate the total cell response for one cluster by

$$V_{\text{cyt}} \bar{\mathbf{c}} = 2\pi\, \mathcal{R}\, \mathcal{Q}\, \vec{\chi}_{lp} + \frac{4\pi b_c^3}{3}\, \vec{\chi}_{00} , \tag{50}$$

what can be used to determine $\bar{e}(t)$ by relations (45) and (46). In the case of no $Ca^{2+}$ conservation, $\bar{e}(t)$ is given by

$$\bar{e}(t) = \bar{e}_0 - \gamma \int_0^t \left[\sigma(t') - \bar{c}(t')\sigma_p + \sigma_l\left(\bar{e}(t') - \bar{c}(t')\right)\right] dt' , \tag{51}$$

this means by the difference of the initial ER concentration $\bar{e}_0$ and the difference of the released $Ca^{2+}$ and $Ca^{2+}$ pumped back into the ER. This method requires the calculation of the average cytosolic $Ca^{2+}$ concentration as well.

The real concentrations are obtained by rescaling $\bar{c}(t)$ and $\bar{e}(t)$ according to Equation (7).

# 4 Green's cell algorithm implementation

The analytical solution for the concentration dynamics (40) can be used as a natural environment for localized $IP_3R$ clusters to study the interplay of their nonlinear stochastic opening behavior and the feedback of $Ca^{2+}$. The stochastic opening and closing of the $IP_3Rs$ is translated by the single channel approximation to time-dependent source terms in the RDS (see Box 1 in the main text). The stochastic transitions depend on the local $IP_3$ and $Ca^{2+}$ concentrations and are modelled by a hybrid version of the Gillespie algorithm [7], which was already used by Rüdiger *et al.* in relation to $Ca^{2+}$ dynamics [8].

## 4.1 Gillespie algorithm

The Gillespie algorithm allows for simulation of stochastic processes [9]. Given the actual time $t$, the probability that the next stochastic event occurs in the infinitesimal time interval $[t+\tau, t+\tau+dt]$ and is an event $\Xi_i$, is given by

$$P(\tau, i)dt = \alpha_i e^{-\alpha_0 \tau} dt \ , \tag{52}$$

where $\alpha_0 = \sum \alpha_j$ is the sum of all propensities. The event probability $P(\tau, i)$ can be realized by drawing two random numbers $r_1$ and $r_2$ from a uniform distribution in the interval $[0, 1]$. Then $\tau$ and $i$ are determined by

$$\alpha_0 \tau = \ln\left(1/r_1\right) \ , \quad \sum_{j=1}^{i} \alpha_j \leq \alpha_0 \, r_2 < \sum_{j=i+1}^{n} \alpha_j \ . \tag{53}$$

The original Gillespie method assumes that the propensities during two events stay constant. This is not valid for our problem, since opening and closing of channels change the $Ca^{2+}$ concentration respectively the propensities by up to three orders of magnitudes.

To resolve this problem we use the method described in [8], which adopt the hybrid version of the Gillespie algorithm [7] to $Ca^{2+}$ dynamics. The time of the next stochastic

event is determined by solving

$$\int_t^{t+\tau} \alpha_0(s,c)ds = \ln(r_1) \ , \tag{54}$$

where the propensities $\alpha_0$ depends explicitly on time $s$ and the concentration $c$. For constant $\alpha_0$ Equation (54) simplifies to (53). For the determination of $\tau$ we rewrite the first equation in (53) by introducing a new variable $g(\tau)$ and solve

$$\dot{g}(s) = \alpha_0(s,c) \ , \tag{55}$$

with the initial condition $g(0) = 0$. A reaction occurs, when $g(s)$ reaches $\ln(r_1)$. The corresponding reaction event is determined as before by a second random number $r_2$ according to the second condition in Equation (53).

For the $Ca^{2+}$ dynamics, the channel transitions $\Xi$ correspond to jumps on the Markov chains representing the subunits of the DKM shown in Supporting Figure 1A. Hence, $\Xi$ describes the transition $X_{ijk} \rightarrow X_{i'j'k'}$, where a single transition does only change one index. For a channel consisting of four subunits the $X_{ijk}$ correspond to occupation numbers. Since each of the four subunit is in one of the states the sum over all $i$, $j$ and $k$ equals 4. In this context $\Xi$ corresponds to an decrease of $X_{ijk}$ by 1 and a subsequently increase of another state $X_{i'j'k'}$ by 1 according to Supporting Figure 1A.

Since the $Ca^{2+}$ concentrations at open channel clusters are rather high, the $Ca^{2+}$ concentration dependent transitions are favored at open clusters. This leads to small time steps. Moreover, the $Ca^{2+}$ concentration changes due to channel transitions very rapidly, and consequently we have to calculate the concentrations for many times.

### 4.2 Parallel algorithm structure (Supporting Figure 4)

To deal with this enormous computational requirements, we developed a parallel algorithm in C++ using the standard library for parallel computing *Message Passing Interface* (MPI) and the *Gnu Scientific Library* (GSL) for the implementation of Bessel functions $J_{l+1/2}(x)$ and Legendre polynomials $P_l(\cos\theta)$. The two key elements of the algorithm are

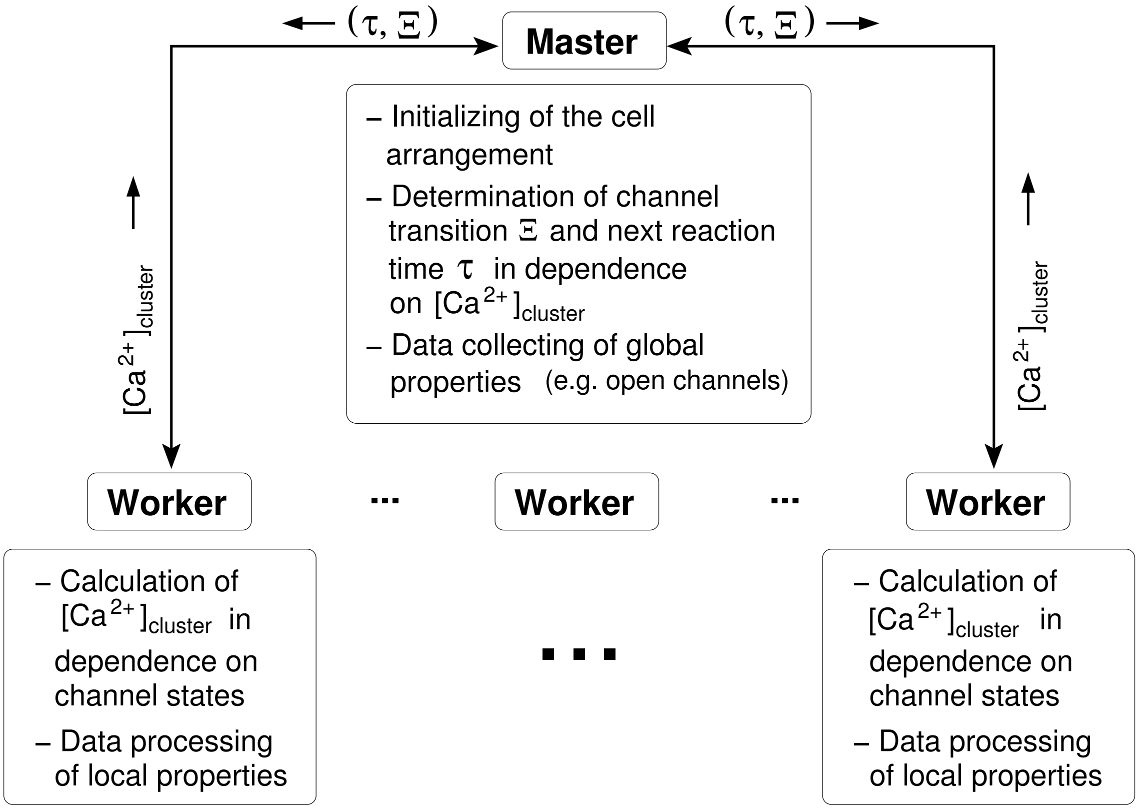


Supporting Figure 4: The scheme of the parallel Green's cell model algorithm. The algorithm is split into two parts. The master process determines the cell arrangement and performs the Gillespie algorithm leading to channel transitions $\Xi$ and reaction times $\tau$. Therefore it relies on the $Ca^{2+}$ concentrations $[Ca^{2+}]_{\text{cluster}}$ at the cluster locations, which are calculated by the analytical solution on worker processes in dependence on the channel state history $\sigma_i(t)$ of each cluster.

the determination of channel transitions and the calculation of the analytical concentration responses at the cluster locations. One benefit of the analytical solution of the linear RDS is that we only have to calculate the concentration at points where $IP_3R$ clusters are localized. Another advantage is the linearity, which enables us to calculate the concentrations of clusters independently and to superpose them in the following.

These properties can be used for a sufficiently parallelized algorithm depicted in Supporting Figure 4. Therefore the problem is split into two parts leading to two different kinds of processes. The *master* process is the "coordinator" of the algorithm, which determines the channel transitions, reaction times and collects global properties. For this

tasks it requires the concentration at the $IP_3R$ clusters. These are determined by *worker* processes, which calculate the concentration at a cluster according to Equation (40).

The $Ca^{2+}$ concentration of each cluster is calculated by a single worker process for which the channel state history of all clusters is required. Therefore each worker process has a copy of the source term $\sigma_j(t)$ of each cluster. These source vectors are updated according to the Gillespie algorithm performed by the master process. This relies on the $Ca^{2+}$ concentration at each cluster. Hence, in each iteration step one bidirectional communication occurs. First the master process broadcasts the time of the next reaction $\tau$ and the corresponding channel transition $\Xi$ consisting of three integers: which reaction occurs in which channel of which cluster. Second each worker process sends the calculated $Ca^{2+}$ concentration of the corresponding cluster to the master process, where they are used to determine the next transition $\Xi$ and occurrence time $\tau$.

The structure of the algorithm can be summarized as follows.

- The master process initializes the cell arrangement by reading a parameter file, which determines e.g. the cell radius $R$, the number of clusters $N_{\mathrm{cl}}$ and their locations, the $Ca^{2+}$ base level $\left[Ca^{2+}\right]_0$ and buffer concentrations, buffer dissociation rates and various other properties of the RDS. The clusters are set randomly with a randomly chosen number of channels in the cell. The channel states are initialized according to the stationary probabilities defined in Equations (1).

- These specifications are sent to all other processes and translated into dimensionless parameters. Moreover, the $\lambda_{lp}$s and most of the response functions $\chi$ are determined and stored at each worker process with the cluster specific values.

- The master determines the next estimated reaction time by relation (54). For the new time $t_{\mathrm{new}} = t_{\mathrm{old}} + \tau$ the property $g_{\mathrm{new}}$ is calculated by (55). Therefore the master collects the $Ca^{2+}$ concentrations at each cluster $[Ca^{2+}]_{\mathrm{cluster}}$ from the corresponding worker processes.

- If $g_{\mathrm{new}} < \ln r_1$ (no stochastic event occurs), the master sets $t_{\mathrm{old}} = t_{\mathrm{new}}$ and determines the next time step $\tau$, which is broadcasted to the worker processes.

- If $g_{\mathrm{new}} \geq \ln r_1$ (a stochastic event occurs in $[t_{\mathrm{old}} = t_{\mathrm{new}}]$), the event time $t_s$ and the corresponding $[\mathrm{Ca}^{2+}]_{\mathrm{cluster}}$ are determined by linear interpolation. The master process draws a random number $r_2$ and determines the stochastic event $\Xi$ according to Equation (53) and updates the channel states. The next time step $\tau$ is determined and sent together with the channel transition $\Xi$ to the worker processes. Then $g_{\mathrm{new}} = 0$ is set and a new random number $r_1$ is drawn on the master process.

# 5 Spiking in dependence on $IP_3$ and $Ca^{2+}$ (Supporting Figure 5)

To analyze the dependence of spiking on the $IP_3$ and $Ca^{2+}$ base level concentrations, we used a fixed setup of a cell with 47 randomly distributed clusters, which are separated by a minimal cluster distance of 1.4 μm and have between 4 and 16 channels each. The standard parameters of the RDS are given in Table 1 of the main text.

The prototype of a cell was simulated with different values of the cytosolic $Ca^{2+}$ base level and $IP_3$ concentration leading to distinct channel behaviors as shown in Supporting Figure 5.

If both concentrations are high as in panel A, the channels do not exhibit a cooperative signal, since due to high $\left[Ca^{2+}\right]_0$ most channels are inhibited, and as soon as they are excitable, they will open again and return into the inhibited state leading to the shown noisy signal. This mechanism also holds for very low $[IP_3]$ (D), but now the total amount of sensitized, inhibited and open channels is decreased.

If we switch $\left[Ca^{2+}\right]_0$ to physiological concentrations at high $[IP_3]$, i.e. going from A to B, we observe very regular oscillations. Due to the high $IP_3$ concentration, most channels are in the excitable state, and as soon as a single $IP_3R$ opens, a global wave travels through the system, synchronizing the inhibition of the channels and terminating the $Ca^{2+}$ release. As soon as the inhibiting $Ca^{2+}$ dissociates from the corresponding binding site, one channel will open again since $\left[Ca^{2+}\right]_0$ respectively the open probability is high.

For a further decrease of $\left[Ca^{2+}\right]_0$, means going to B and F, the oscillations become slower and more irregular, as the probability of an initial event decreases (note the different time ranges for each column in Supporting Figure 5). In both cases (B and F) only rare single events, as blips or puffs, are observed, caused by full inhibition and additionally by low $\left[Ca^{2+}\right]_0$ in panel F.

Lowering the $IP_3$ concentration for fixed $\left[Ca^{2+}\right]_0$, i.e. going from B to C, H and E or from F to G, K and J, causes an increase of $T_{av}$ and shrinks the amplitudes, as the channels are less sensitized and the nucleation probability decreases. In these less sensitized

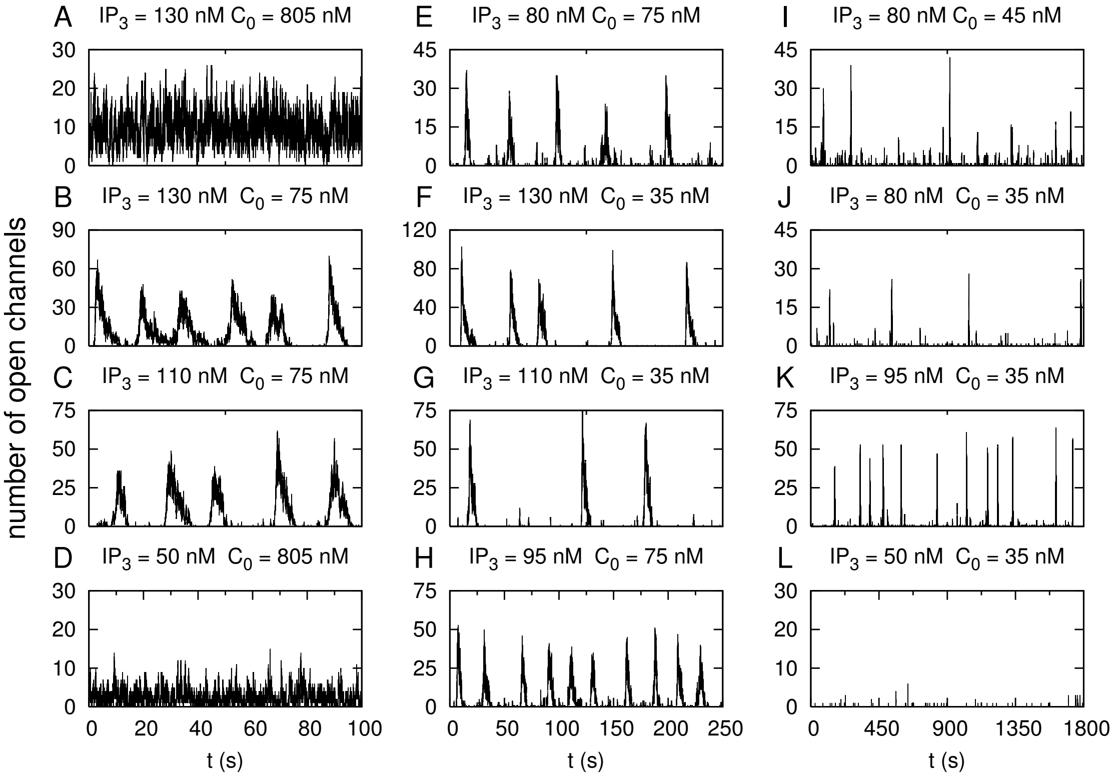


Supporting Figure 5: Channel signals of a specific cell with 47 randomly scattered clusters having between 4 and 16 channels for different $Ca^{2+}$ base level concentrations $[Ca^{2+}]_0$ and $IP_3$ concentrations $[IP_3]$. For very high (A) and low (L) concentrations, no constructive signal of the total 405 channels is observed, whereas in the intermediate parameter region, the nonlinear properties of the $IP_3R$ combined with those of the RDS serve for fast and regular oscillations (such as in B) or slower irregular oscillations with smaller amplitudes (J). Note the different time ranges for each column and see text for more details.

regimes, $[Ca^{2+}]_0$ sets the probability for the initial events, as can be seen by comparing E, I and J, where puff sizes decrease with decreasing $[Ca^{2+}]_0$, but the amplitudes of the less frequent spikes at $[Ca^{2+}]_0$= 45 nM are similar to those with 75 nM but shrink for a even lower concentration of 35 nM.

Finally, if we go to very low concentrations of both $IP_3$ an $Ca^{2+}$(L), no spikes are observed, and the resulting signal is the random overlay of single uncoordinated blips. From a physiological point of view, oscillations evolve from the last depicted case of low concentrations by increasing $[IP_3]$ caused by external or internal signals inducing PLCs.

# 6 Population slopes (Supporting Figure 6)

The population slopes are averages of individual slopes obtained by single parameter variations. By varying the mobile buffer concentration for a fixed cell, the cell exhibits different $\sigma$-$T_{av}$ values leading to individual $\sigma$-$T_{av}$ relations as those shown in Supporting Figure 6. Each panel shows the relation for a fixed cell with varying buffer concentration and different panels correspond in these representative examples to different minimal intercluster distances. For each cell, the slope is determined by linear regression leading to individual slopes and deterministic recovery periods $T_{det}$ as stated in the panels. These values lead to the population slope $m_{buffer}$ by averaging.

The cells in column A have a single channel current of 0.12 pA and a BAPTA concentration varying between 5 µM and 35 µM. The minimal intercluster distance decreases from top to bottom from 2 µm to 1.5 µm and 1 µm. Smaller distances lead to smaller $\sigma$ and $T_{av}$ values but the individual slopes are all close to 0.9 and also $T_{det}$ is in the same range.

Cells in column B have a single channel current of 1.2 pA, a BAPTA concentration between 5 µM and 125 µM and decreasing intercluster distances from 2.5 µm to 2 µm and 1.5 µm. Smaller distances again lead to smaller values of $\sigma$ and $T_{av}$. The individual slopes are rather self-consistent and decreased to 0.6 compared to the cells with the smaller current.

Analogously, we determined also individual slopes for cells which do not vary in the buffer concentration but in the $IP_3$ concentration (between 60 nM and 0.3 µM), spatial arrangements (minimal intercluster distances between 1 µm and 2.5 µm) and pump strength (between 22 $s^{-1}$ and 250 $s^{-1}$). It turns out that the observed dependence of the slope on the current strength is stable under these circumstances as well. This points to a functional robustness as explained in the main text.

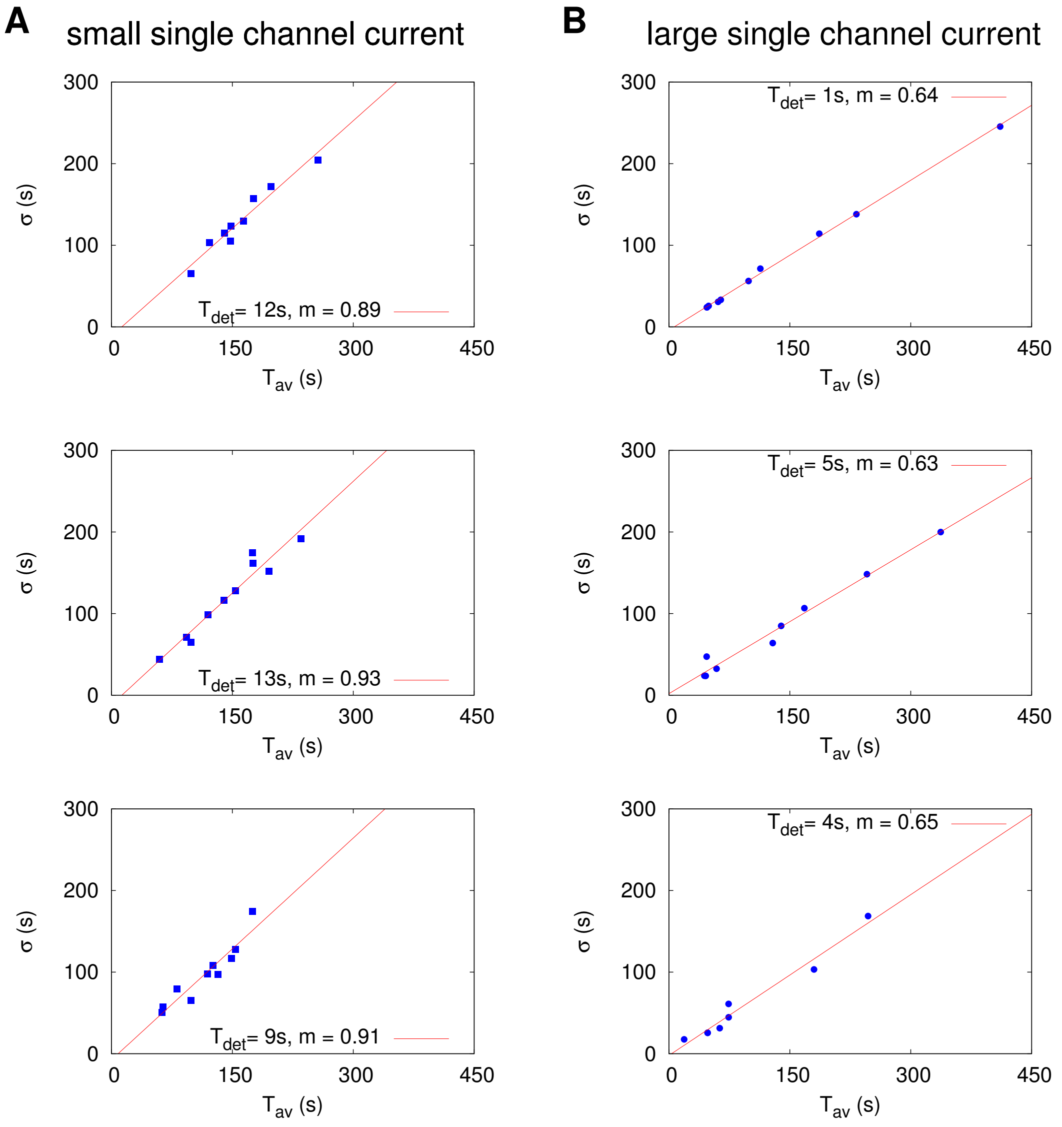


Supporting Figure 6: Determination of population slope from individual slopes $m$ for small (**A**) and large (**B**) single channel currents. Each panel depicts the $\sigma$ and $T_{av}$ values obtained from a fixed cell with varying buffer concentrations. The slope of the individual $\sigma$-$T_{av}$ relations are obtained by linear regression and the population slope is determined by the average of the individual slopes. The minimal intercluster distance decreases from top to bottom for each column leading to smaller $\sigma$ and $T_{av}$ values. (See text for more details.)

# References

[1] Mak D, McBride S, Foskett J (2003) Spontaneous channel activity of the inositol 1,4,5-trisphosphate ($InsP_3$) receptor ($InsP_3R$). application of allosteric modeling to calcium and $InsP_3$ regulation of the $InsP_3R$ single-channel gating. JGenPhysiol 122: 583–603.

[2] Ramos-Franco J, Fill M, Mignery G (1998) Isoform-specific function of single inositol 1,4,5-trisphosphate receptor channels. BiophysJ 75: 834–839.

[3] Beck J, Cole K, Haji-Sheikh A, Litkouhi B (1992) Heat Conduction Using Green's Function. New York: Series in Computational and physical processes.

[4] Özisik M (1993) Heat conduction. New York: John Wiley and sons.

[5] Morse P, Feshbach H (1954) Methods of theoretical Physics. New York: McGraw-Hill Book Company.

[6] Abramowitz M, Stegun A (1970) Handbook of Mathematical Functions. New York: Dover Publication.

[7] Alfonsi A, Cancès E, Turinici G, Ventura BD, Huisinga W (2004) Exact simulation of hybrid stochastic and deterministic models for biochemical systems. INRIA Rapport de Recherche, Thèmes NUM et BIO 5435.

[8] Rüdiger S, Shuai J, Huisinga W, Nagaiah C, Warnecke G, et al. (2007) Hybrid stochastic and deterministic simulations of calcium blips. Biophys J 93: 1847–1857.

[9] Gillepsie D (1977) Exact stochastic simulation of coupled chemical reactions. J Phys Chem 8: 2340–2354.
